# Supplementary figures and images for: Acetyltransferase Enok regulates transposon silencing and piRNA cluster transcription
Source: PLoS Genet. 2021 Feb 1;17(2):e1009349. doi: 10.1371/journal.pgen.1009349 (PMC7877743; doi:10.1371/journal.pgen.1009349)

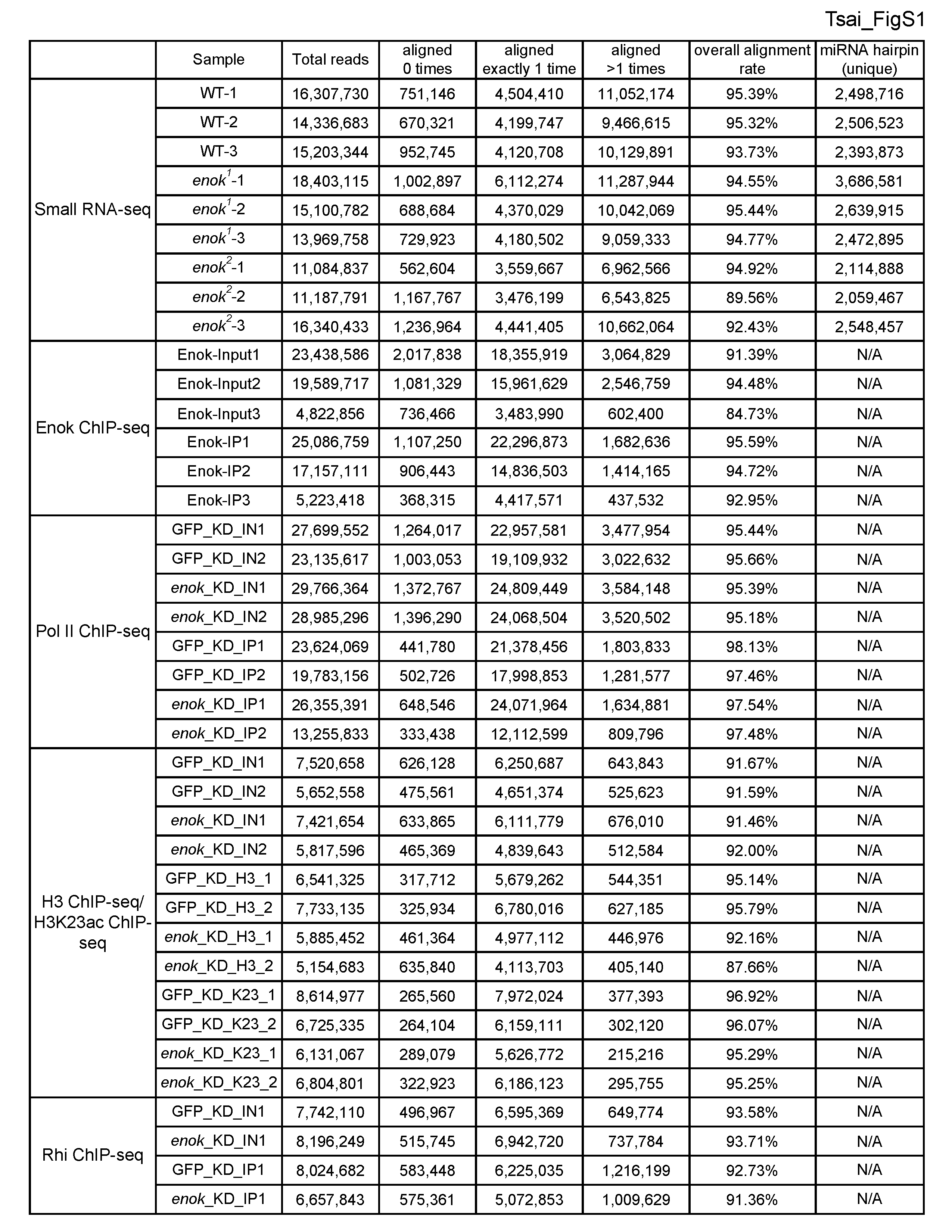

Supplement: S1 Fig — (TIFF) [file pgen.1009349.s001.tiff]

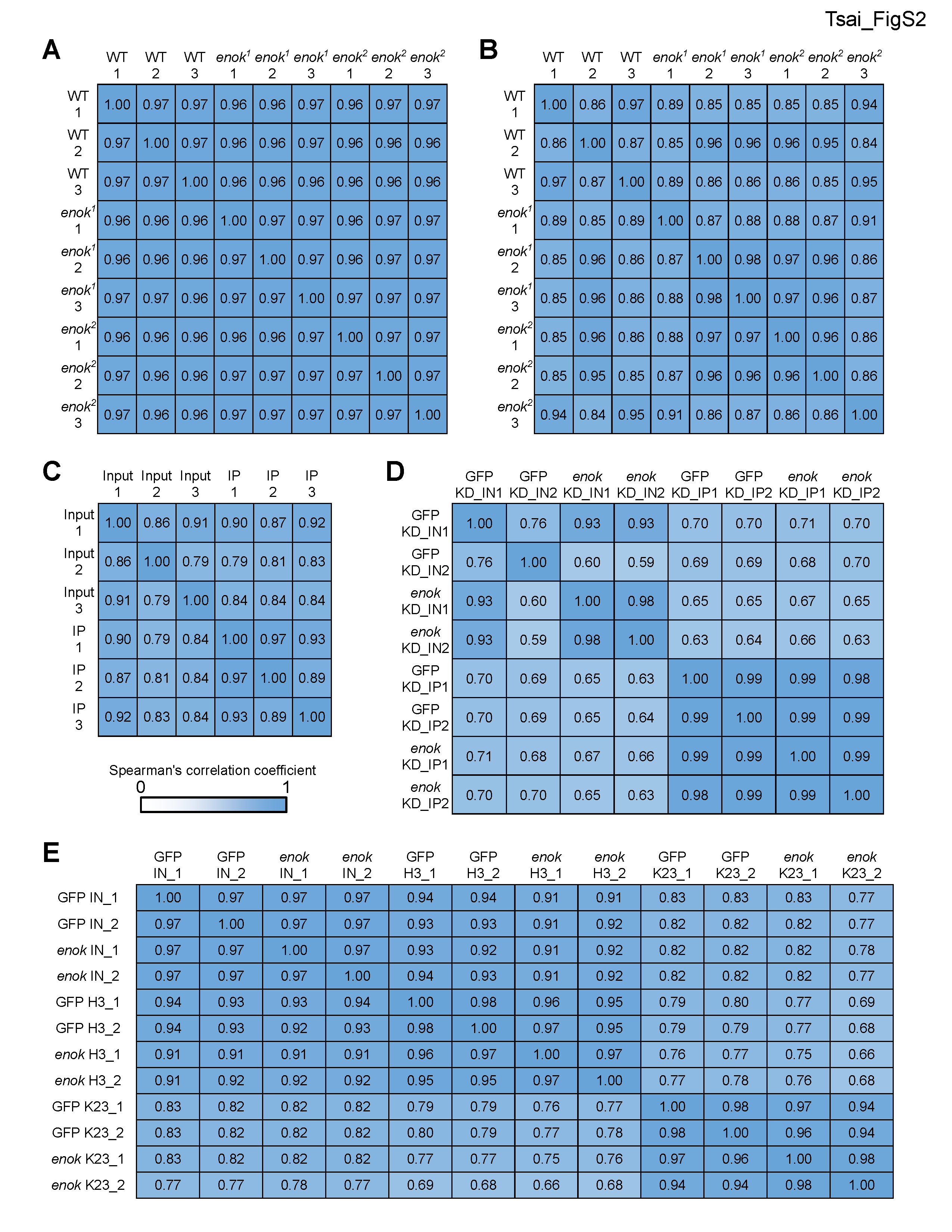

Supplement: S2 Fig — Spearman's correlation coefficients across different samples and replicates in the RNA-seq (A), small RNA-seq (B), Enok ChIP-seq (C), RNA Pol II ChIP-seq (D) and H3K23ac/H3 ChIP-seq (E) analyses were calculated using plotCorrelation from deepTools. The color scale used for all tables is shown in the bottom panel of (C). (TIFF) [file pgen.1009349.s002.tiff]

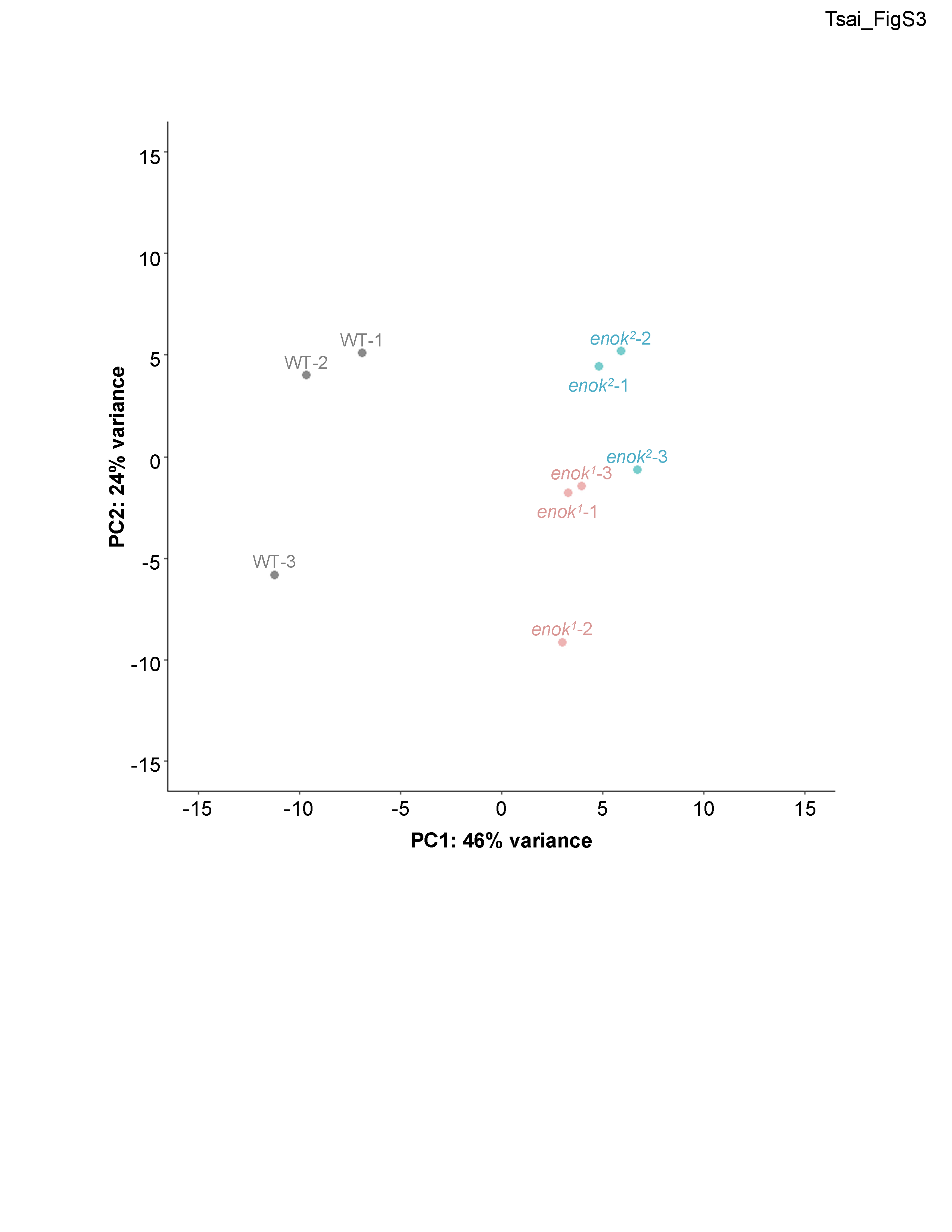

Supplement: S3 Fig — The PCA plot of RNA-seq samples was generated using R. (TIFF) [file pgen.1009349.s003.tiff]

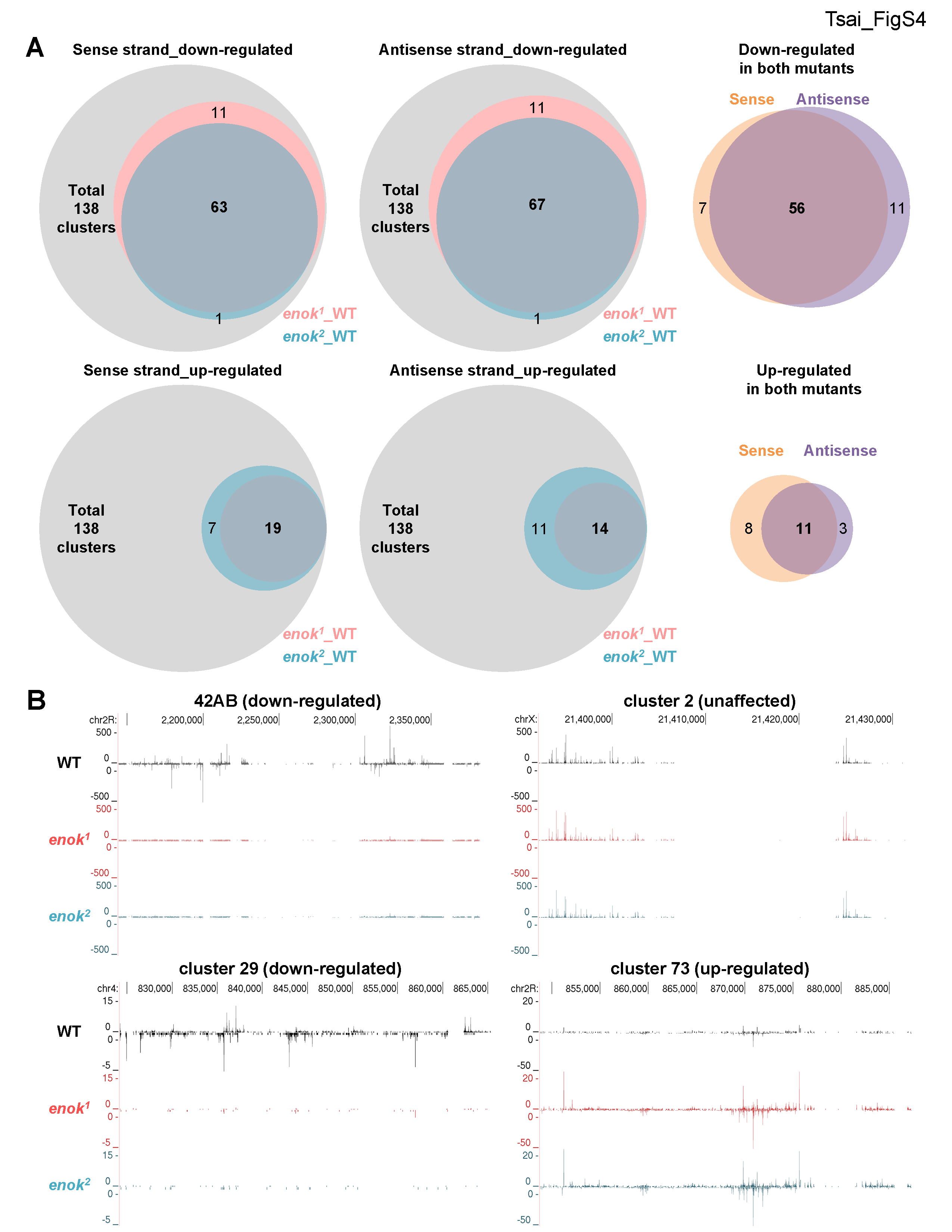

Supplement: S4 Fig — (A) Venn diagrams demonstrating the overlap between the piRNA clusters with different piRNA levels identified by small RNA-seq in the enok1 and enok2 ovaries compared with the WT control. Differential analysis was performed by DESeq2 using the cutoff of adjusted p-value < 0.05. Clusters with down-regulated or up-regulated piRNA levels in mutants compared with the WT control are shown in the top or the bottom panel, respectively. (B) Genome Browser view of small RNA-seq results of four examples of piRNA clusters that either were down-regulated (42AB and cluster 29; left panel), remained unaffected (cluster 2; right top panel) or up-regulated (cluster 73; right bottom panel) in enok mutants as compared with the WT control. In (A-B), genotypes of females are as follows: hs-Flp / +; FRTG13/ FRTG13, ovoD1-18 (WT); hs-Flp / +; FRTG13, enok1/ FRTG13, ovoD1-18 (enok1); hs-Flp/+; FRTG13, enok2/FRTG13, ovoD1-18 (enok2). (TIFF) [file pgen.1009349.s004.tiff]

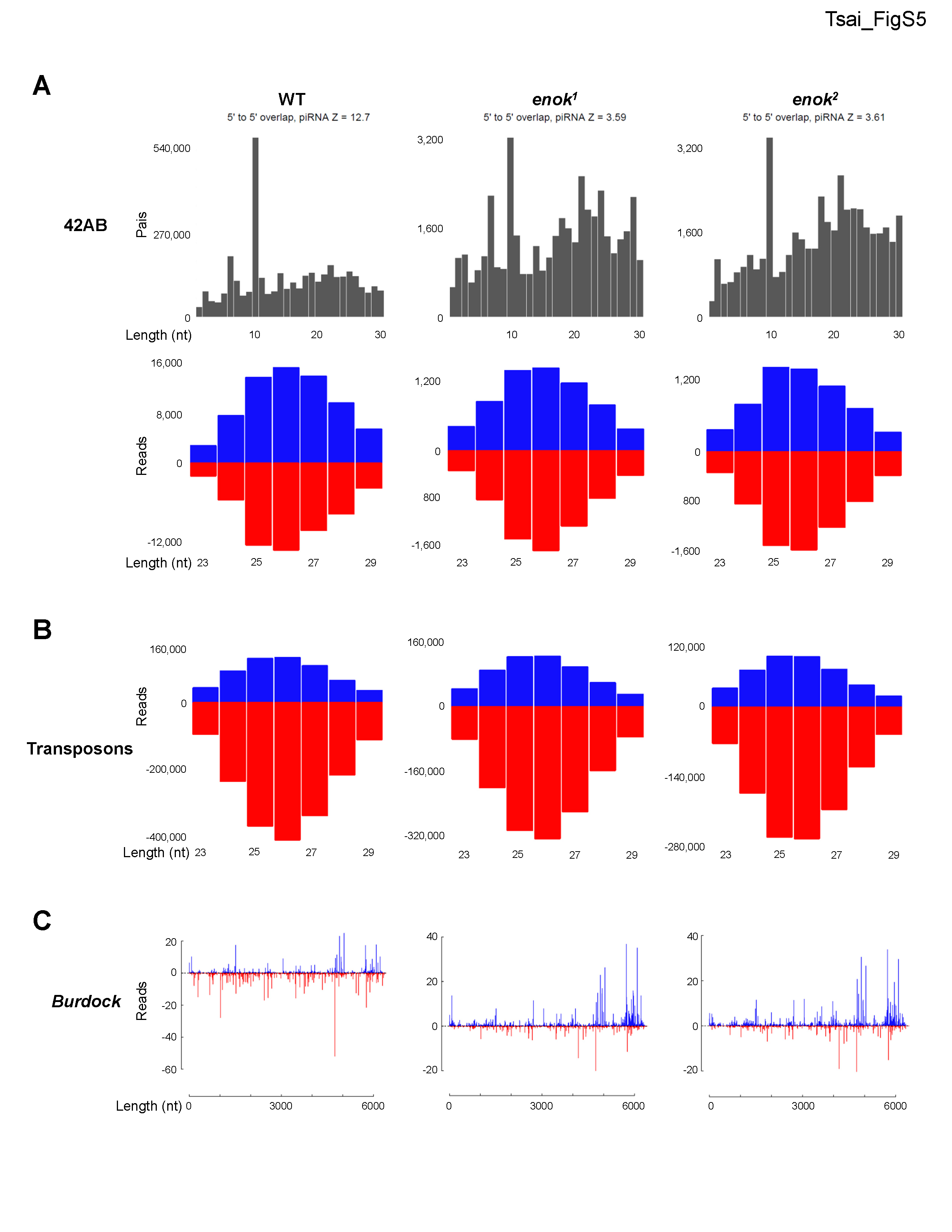

Supplement: S5 Fig — (A) Upper panel: Distribution of overlap sizes between pairs of complementary piRNAs uniquely mapping to 42AB with ping-pong z-scores [36] in the WT and enok mutant germline clone ovaries. Reads of complementary piRNA pairs were normalized to miRNA. Lower panel: Length histograms of piRNAs uniquely mapping to 42AB in the WT and enok mutant germline clone ovaries. Reads were normalized to miRNA. (B) Length histograms of piRNAs mapping to transposons in the WT and enok mutant germline clone ovaries. (C) Sense and antisense piRNA reads mapping to the consensus Burdock sequence. In (A-C), genotypes are as described in S4 Fig. (TIFF) [file pgen.1009349.s005.tiff]

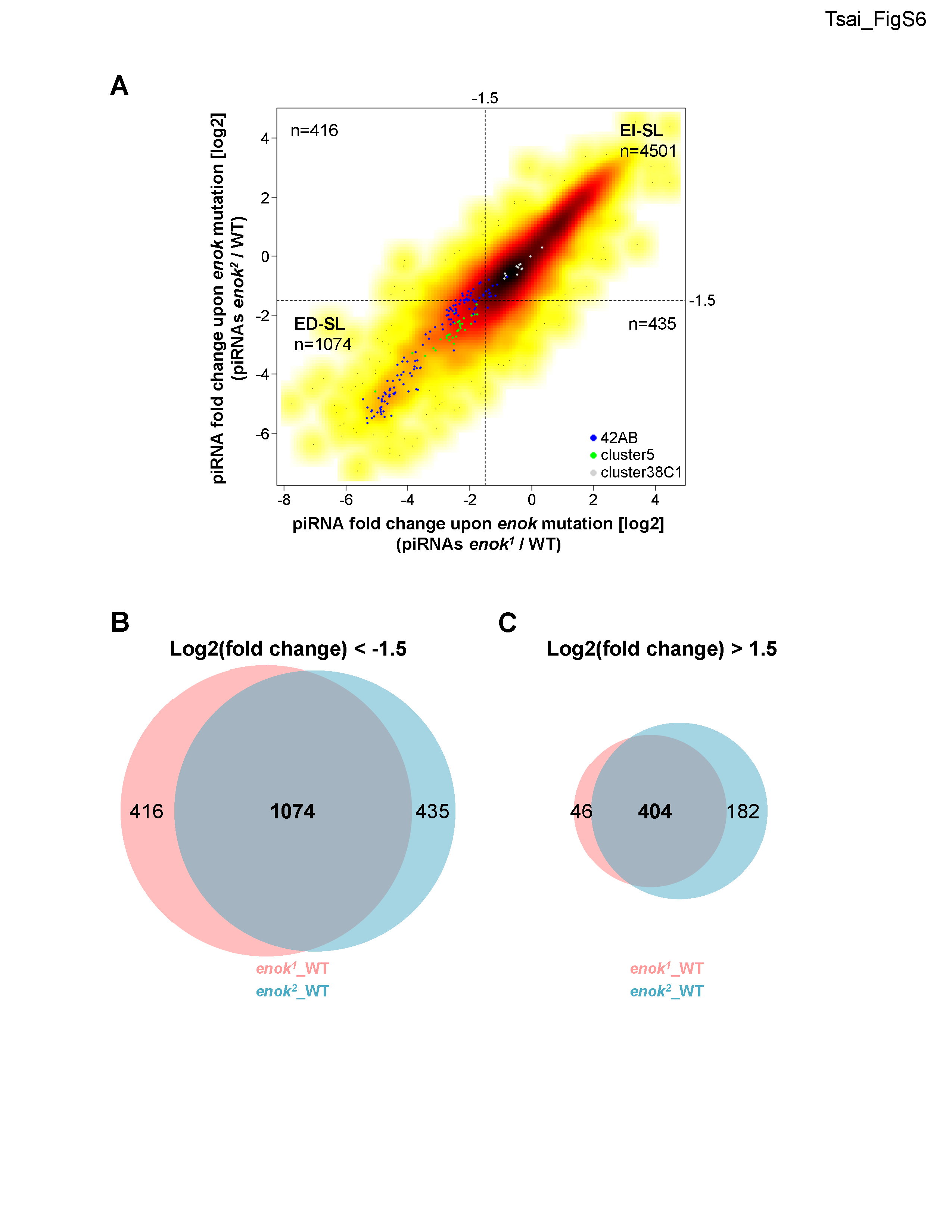

Supplement: S6 Fig — (A) Scatter-plot displaying piRNA fold changes in enok1 (x axis) versus enok2 (y axis) mutants for all Rhi-dependent piRNA source loci (RD-SL). The two classes (ED-SL and EI-SL) and their respective population sizes are indicated. The color gradient (yellow < red < black) indicates the density of underlying 1 kb bins. RD-SL in 42AB, cluster5 or cluster38C1 are indicated using blue, green or grey dots, respectively. (B-C) Venn diagrams demonstrating the overlap between the RD-SL with different piRNA levels identified by small RNA-seq in the enok1 and enok2 ovaries compared with the WT control. RD-SLs with down-regulated or up-regulated piRNA levels in mutants compared with the WT control are shown in (B) and (C), respectively. In (A-C), genotypes are as described in S4 Fig. (TIFF) [file pgen.1009349.s006.tiff]

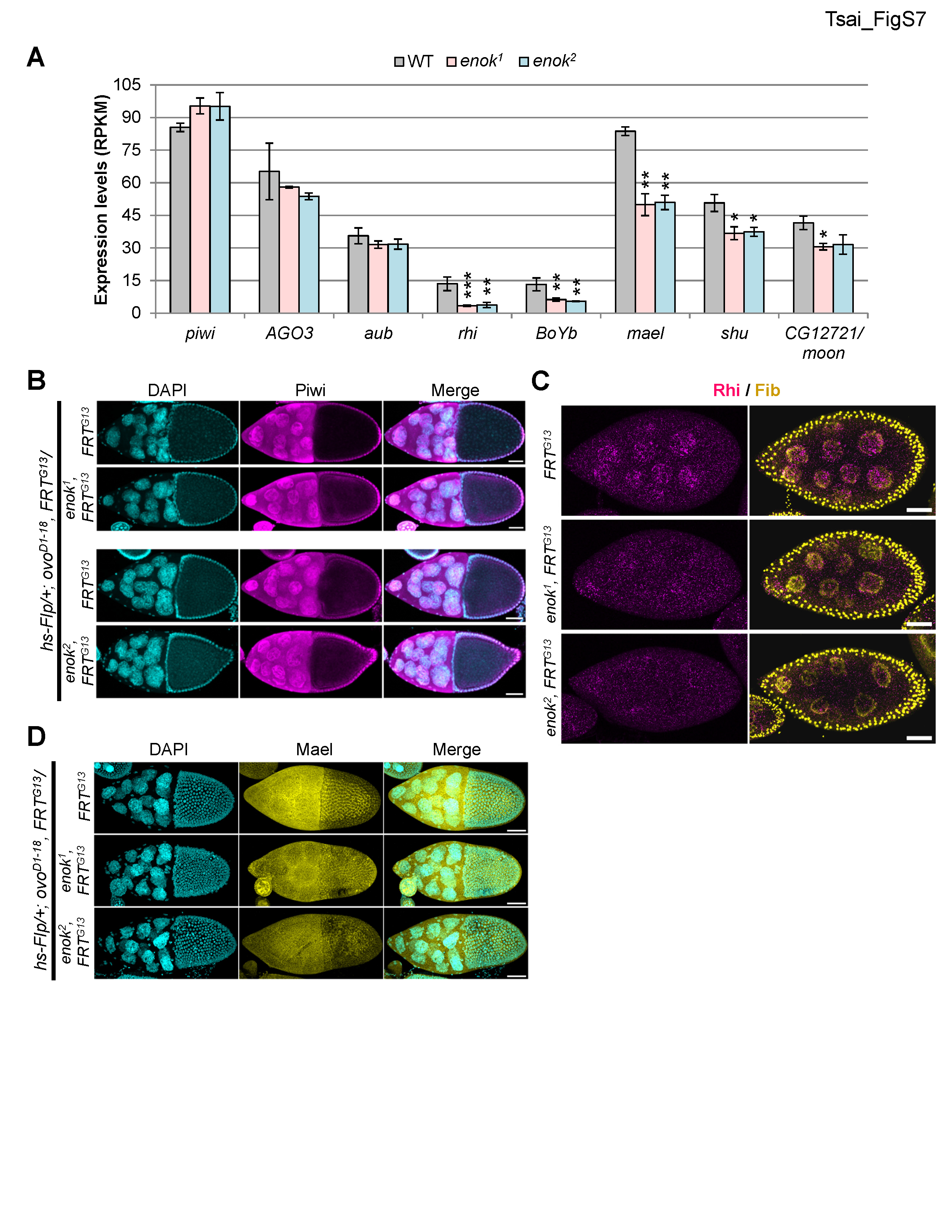

Supplement: S7 Fig — (A) The expression levels of selected genes involved in piRNA biosynthesis in the WT control and enok mutant ovaries are shown in RPKM obtained from RNA-seq (dm3). Data represent the mean of three biological replicates +/- SD. *FDR < 0.05, **FDR < 0.01, ***FDR < 0.001 (edgeR). (B) Stage 10 egg chambers were stained with DAPI and an α-Piwi antibody. Bars: 50μm. (C) Stage 8 egg chambers were stained with α-Rhi and α-Fibrillarin (Fib) antibodies. Projections of 16 sections in the middle of egg chambers are shown. Fib staining is shown as a staining control. Bars: 25μm. (D) Stage 10 egg chambers were stained with DAPI and an α-Mael antibody. Bars: 50μm. Genotypes in (A-D) are as described in S4 Fig. (TIFF) [file pgen.1009349.s007.tiff]

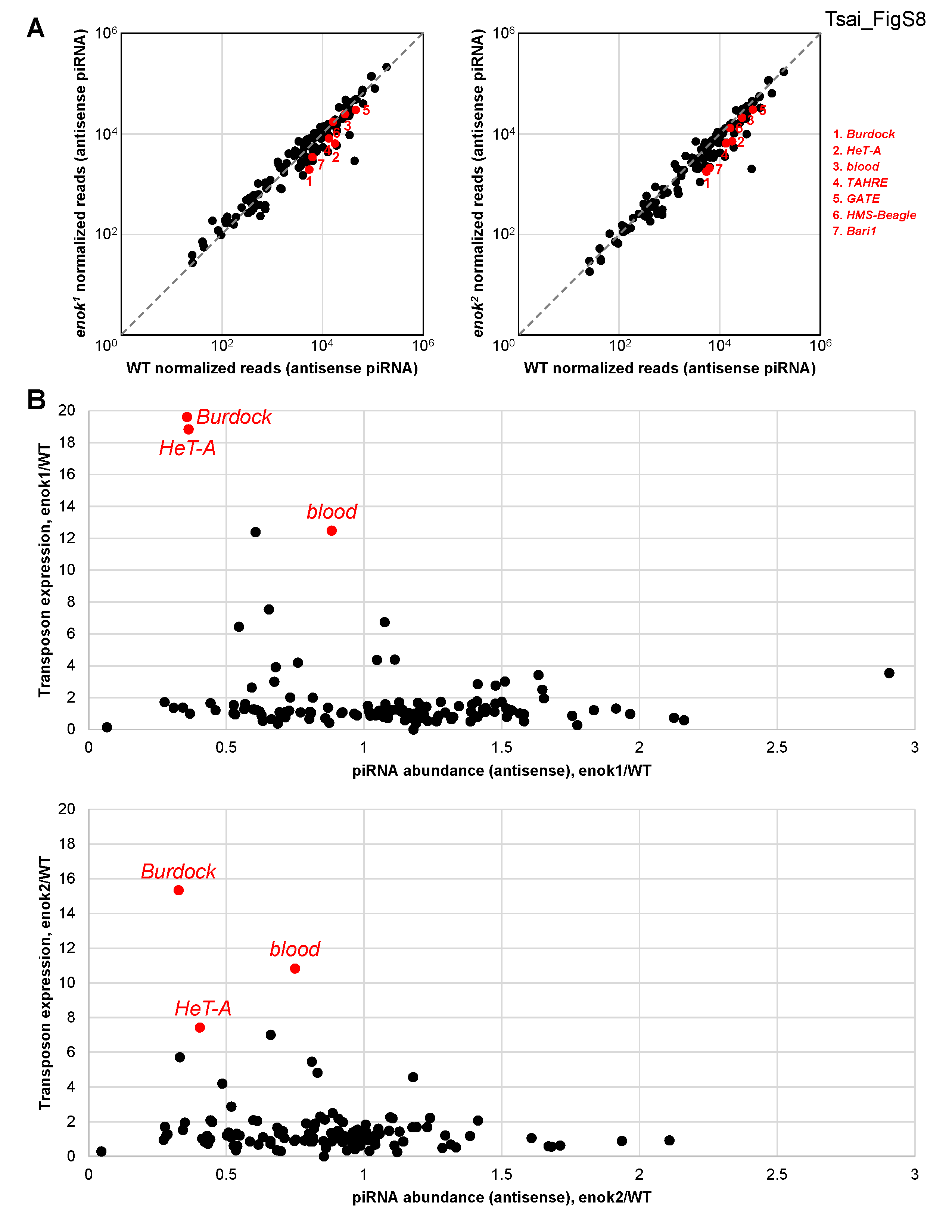

Supplement: S8 Fig — (A) Reads of antisense piRNAs encoded by transposon families (normalized to miRNAs) in two enok mutants are plotted against those in the WT control. The seven transposon families that are significantly activated in enok mutants are labeled by red dots. (B) Fold changes in transposon family expression in two enok mutants (y axis, mutant/WT) plotted against fold changes in antisense piRNAs encoded by the same transposon family (x axis, mutant/WT) are shown. The highly over-expressed transposons (Burdock and HeT-A) also showed large reductions in antisense piRNAs. However, expression of blood increased 10–12 fold while the total antisense piRNA pool was only reduced by 12%-25%. In (A-B), genotypes are as described in S4 Fig. (TIFF) [file pgen.1009349.s008.tiff]

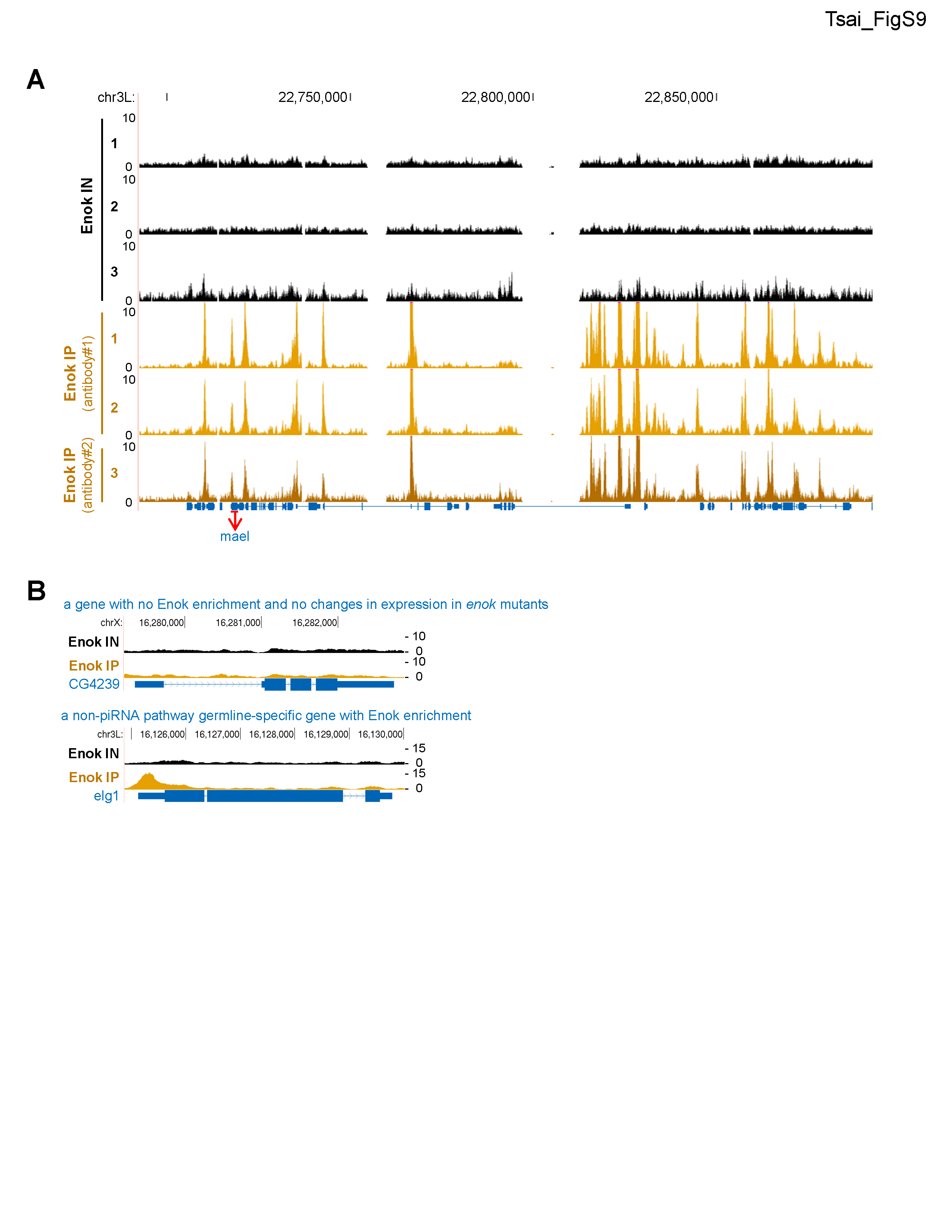

Supplement: S9 Fig — (A) Enok peaks across a 200 kb region, including the mael gene, were reproducibly detected in 3 independent replicates of ChIP-seq analysis. (B) Genome Browser view of Enok ChIP-seq data at CG4239 and elg1. CG4239 represents a negative control with no Enok enrichment and no changes in expression in enok mutants, and elg1 represents a non-piRNA pathway germline-specific gene enriched by Enok. Experimental details are as described in the legend for Fig 3B. (TIFF) [file pgen.1009349.s009.tiff]

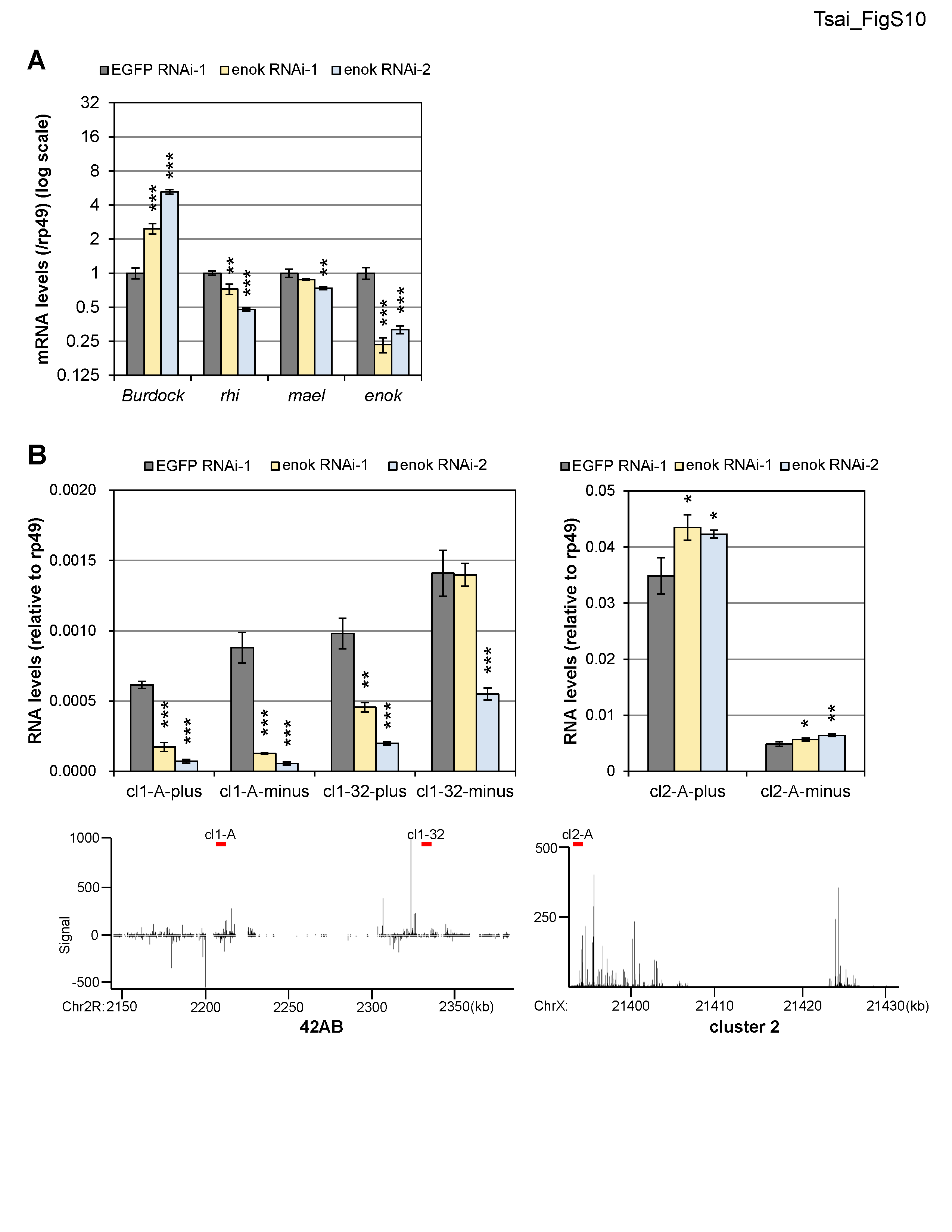

Supplement: S10 Fig — (A) RT-qPCR analysis of ovaries was used to examine the expression levels of the indicated transposon and genes. The mRNA levels were normalized to the levels of rp49. (B) The same total RNA samples used in (A) were subjected to strand-specific RT-qPCR analysis for RNAs derived from 42AB (left panel) and cluster 2 (right panel). The bottom panel shows the signals of piRNA reads mapping to the indicated clusters in WT ovaries as described in Fig 2. Red bars in the bottom panel indicate the location of amplicons used in the qPCR reaction. In (A-B), data represent the mean of three biological replicates +/- SD. *P < 0.05, **P < 0.01, ***P < 0.001 (Student's t-test). Genotypes of the females are as follows: P{w[+mC] = otu-GAL4::VP16.R}1 / +; P{w[+mC] = GAL4-nos.NGT}40 / +; P{w[+mC] = GAL4::VP16-nos.UTR}CG6325MVD1 / P{VALIUM20-EGFP.shRNA.1}attP2 (EGFP RNAi-1); P{w[+mC] = otu-GAL4::VP16.R}1 / +; P{w[+mC] = GAL4-nos.NGT}40 / +; P{w[+mC] = GAL4::VP16-nos.UTR}CG6325MVD1 / P{TRiP.HMS02634}attP2 (enok RNAi-1); P{w[+mC] = otu-GAL4::VP16.R}1 / +; P{w[+mC] = GAL4-nos.NGT}40/+; P{w[+mC] = GAL4::VP16-nos.UTR}CG6325MVD1/P{TRiP.HMS02048}attP2 (enok RNAi-2). (TIFF) [file pgen.1009349.s010.tiff]

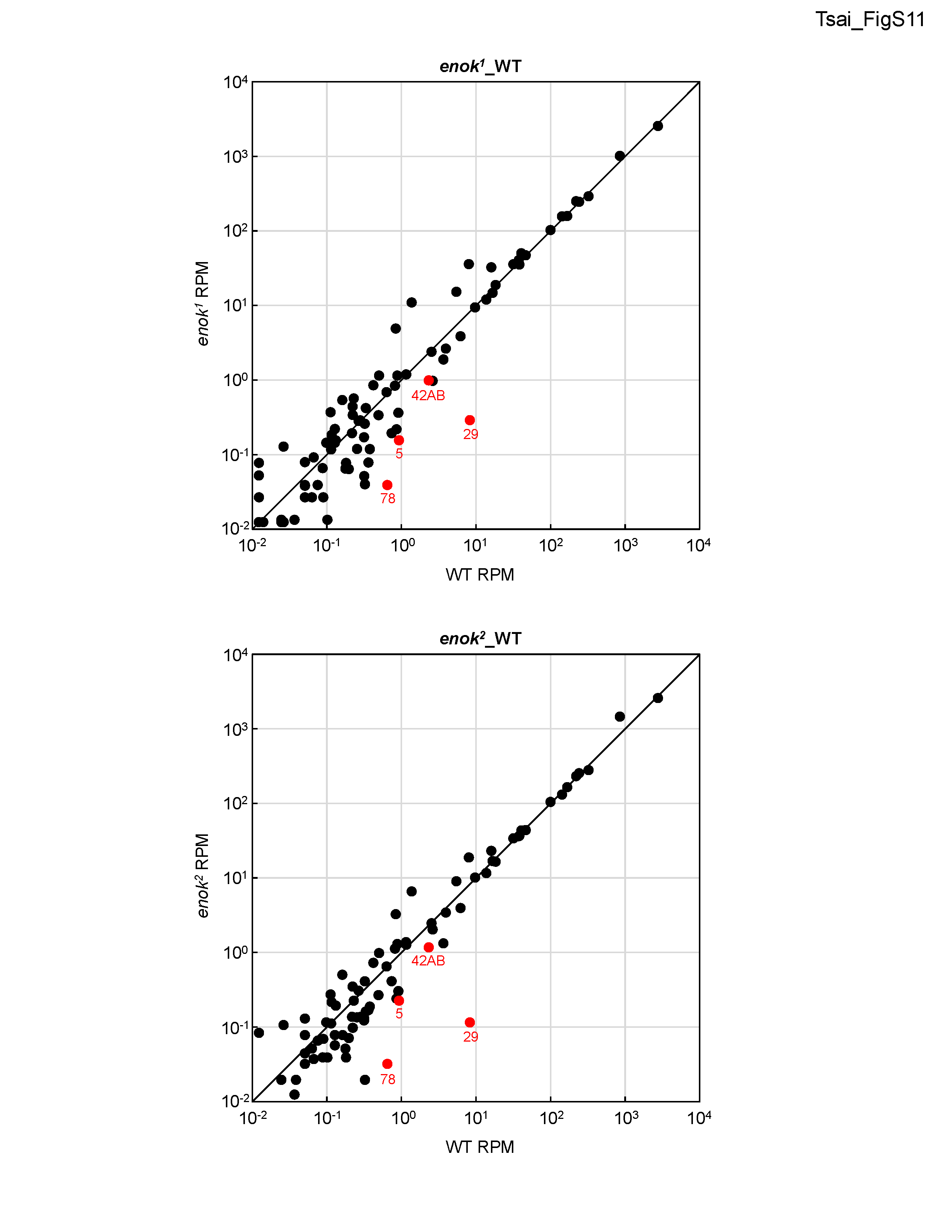

Supplement: S11 Fig — The levels of poly-A selected RNAs that uniquely mapped to piRNA clusters in enok mutant germline clone ovaries versus the WT control are plotted in a log scale. Selected piRNA clusters that were significantly down-regulated in enok mutants are indicated by red dots. Genotypes are as described in S4 Fig. (TIFF) [file pgen.1009349.s011.tiff]

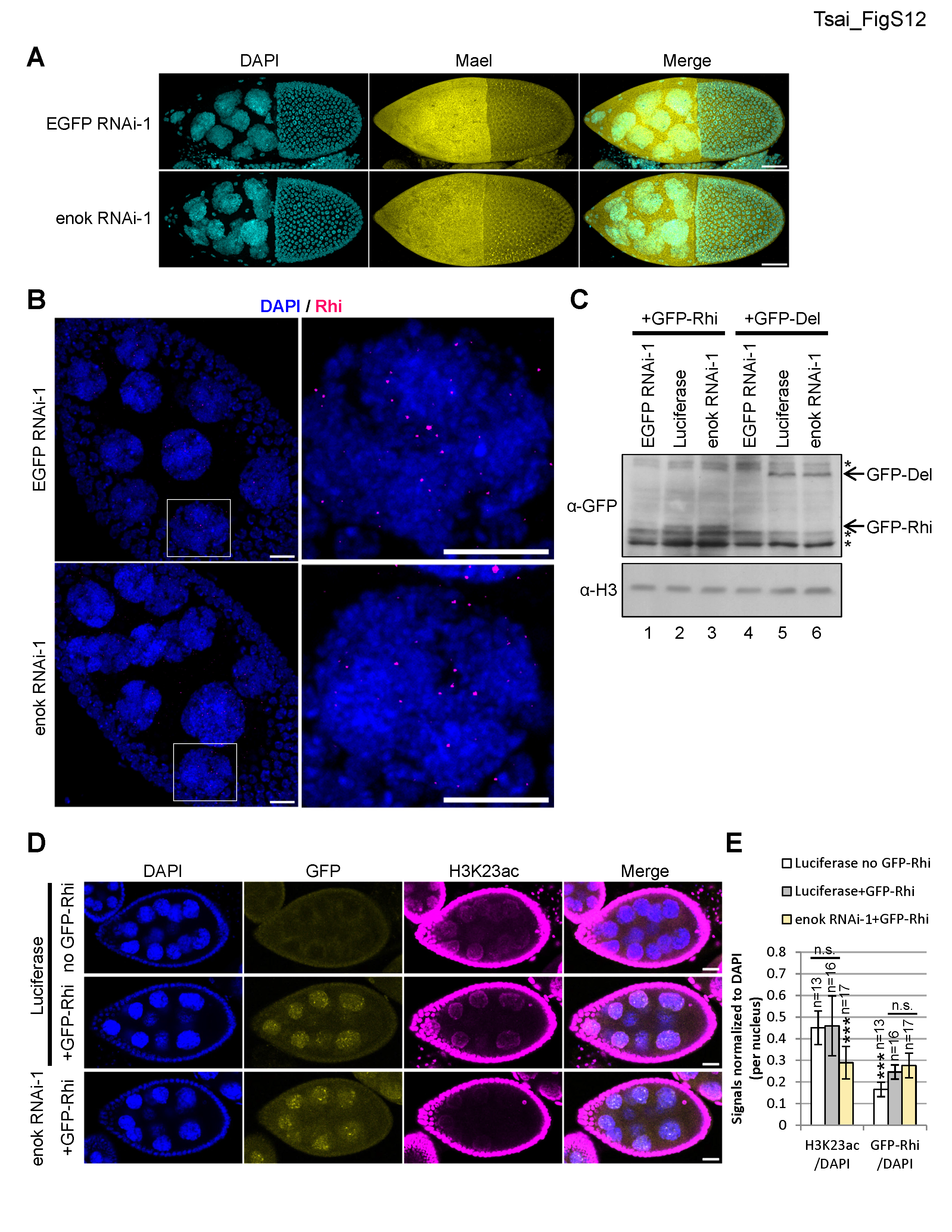

Supplement: S12 Fig — (A) Stage 10 egg chambers were stained with DAPI and an α-Mael antibody. Bars: 50μm. (B) Stage 7 egg chambers were stained with DAPI and an α-Rhi antibody. Left panel: projections of 32 sections in the middle of egg chambers are shown. Right panel: enlarged images of the entire nuclei indicated by white solid boxes in the left panel are shown. Bars: 10μm. (C) Whole cell extracts were prepared from ovaries and subjected to western blotting. H3 was used as the loading control. The EGFP RNAi-1 knockdown, which targets the GFP-tag of GFP-Rhi and GFP-Del, was used as a negative control. Asterisks indicate non-specific bands detected by the α-GFP antibody. (D-E) Stage 8 egg chambers were stained with DAPI and an α-H3K23ac antibody. Representative images are shown in (D). Bars: 20μm. The staining signals of H3K23ac or the GFP signals in the nucleus of nurse cells were quantified and normalized to the DAPI signals. The normalized quantitation results are shown in (E). Data represent the mean +/- SD. All P-values between Luciferase+GFP-Rhi and other ovaries less than 0.001 are indicated using three (***) asterisks (Student's t-test). n.s.: not significant. In (A-B), genotypes are as described in S10 Fig. In (C-E), genotypes are as follows: P{w[+mC] = otu-GAL4::VP16.R}1 / +; P{w[+mC] = GAL4-nos.NGT}40 / +; P{w[+mC] = GAL4::VP16-nos.UTR}CG6325MVD1, P{rhi-GFP} / P{VALIUM20-EGFP.shRNA.1}attP2 (EGFP RNAi-1+GFP-Rhi); P{w[+mC] = otu-GAL4::VP16.R}1 / +; P{w[+mC] = GAL4-nos.NGT}40 / +; P{w[+mC] = GAL4::VP16-nos.UTR}CG6325MVD1, P{rhi-GFP} / P{UAS-LUC.VALIUM10}attP2 (Luciferase+GFP-Rhi); P{w[+mC] = otu-GAL4::VP16.R}1 / +; P{w[+mC] = GAL4-nos.NGT}40 / +; P{w[+mC] = GAL4::VP16-nos.UTR}CG6325MVD1, P{rhi-GFP} / P{TRiP.HMS02634}attP2 (enok RNAi-1+GFP-Rhi); P{w[+mC] = otu-GAL4::VP16.R}1 / +; P{w[+mC] = GAL4-nos.NGT}40 / +; TM3,Ser/ P{UAS-LUC.VALIUM10}attP2 (Luciferase; no GFP-Rhi); P{w[+mC] = otu-GAL4::VP16.R}1 / +; P{w[+mC] = GAL4-nos.NGT}40 / +; P{w[+mC] = GAL4::VP16-nos.UTR}CG6325MVD1, P{ [file pgen.1009349.s012.tiff]

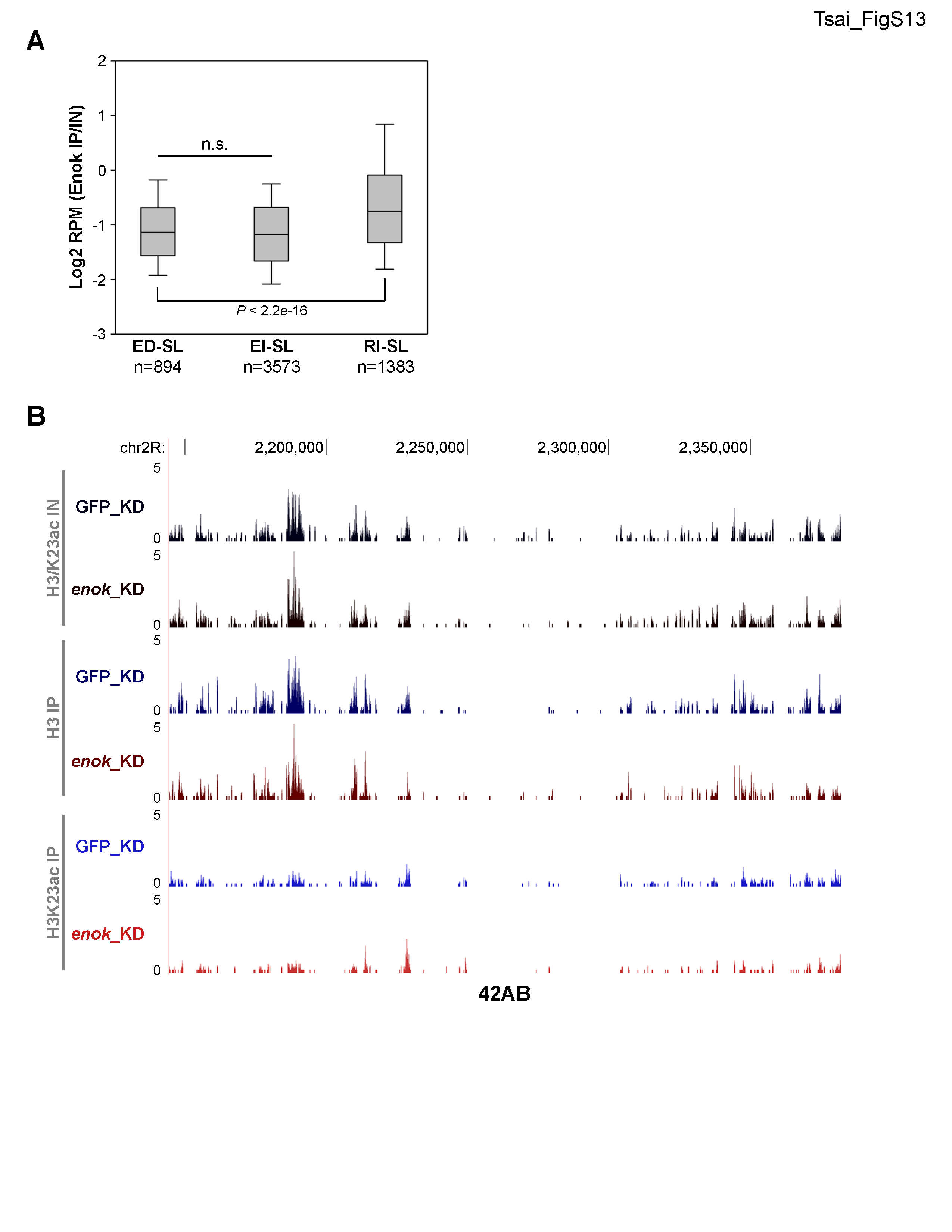

Supplement: S13 Fig — (A) A box-plot displaying the distribution of Enok occupancies for all 1 kb bins belonging to the indicated groups. Center line, median; box limits, upper and lower quartiles; whiskers, the 5th and 95th percentile (outliers not shown). P-values were calculated using Wilcoxon Rank sum test. n.s.: not significant. (B) Genome Browser view of H3K23ac and H3 ChIP-seq data at 42AB. ChIP-seq experiments were performed in two independent replicates, and results from one representative replicate are shown. Input (IN) and immunoprecipitation (IP) tracks are shown. Genotypes of the females are as follows: tj-GAL4/+; P{w[+mC] = GAL4::VP16-nos.UTR}CG6325MVD1 / P{VALIUM20-EGFP.shRNA.1}attP2 (GFP_KD); tj-GAL4/+; P{w[+mC] = GAL4::VP16-nos.UTR}CG6325MVD1/P{TRiP.HMS02634}attP2 (enok_KD). (TIFF) [file pgen.1009349.s013.tiff]

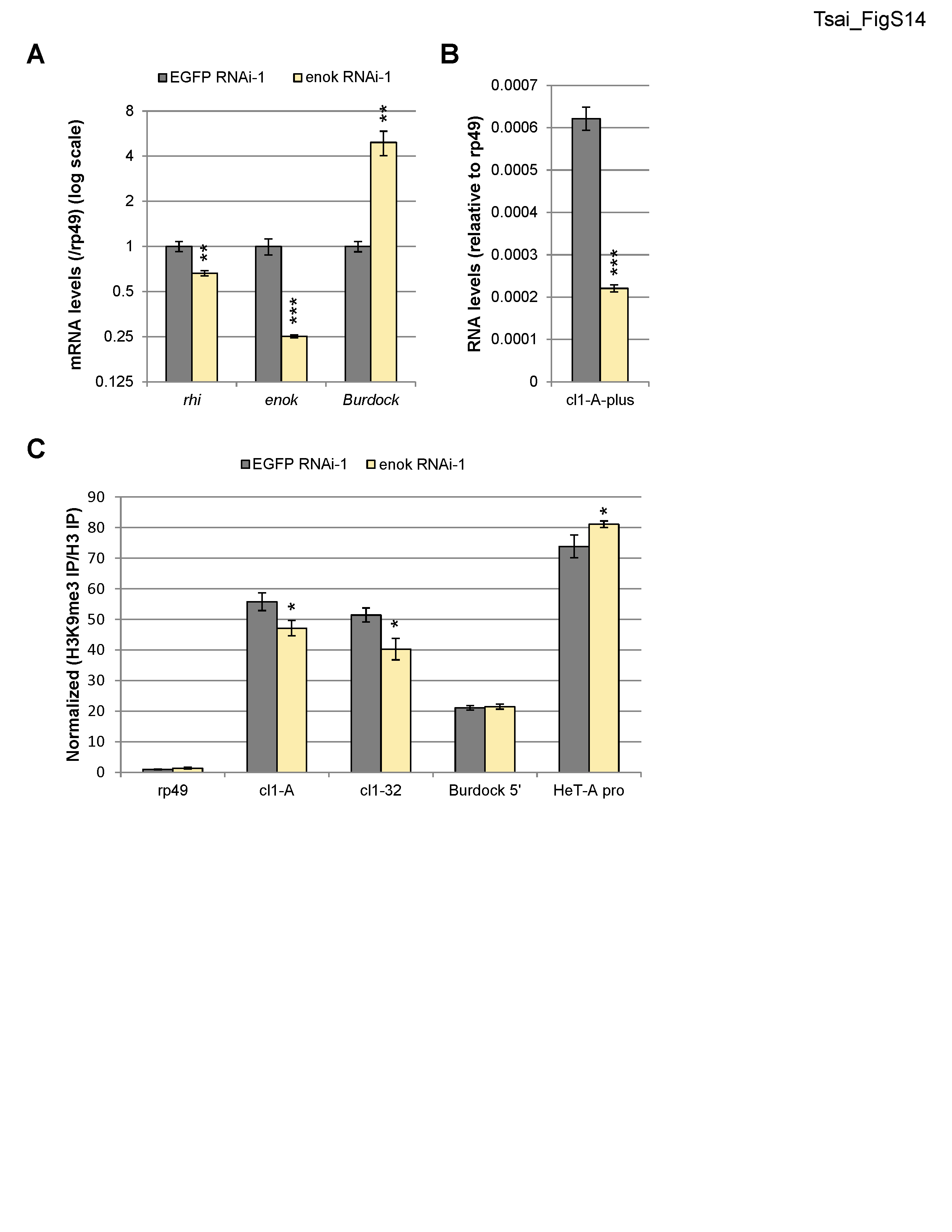

Supplement: S14 Fig — (A) RT-qPCR analysis of ovaries was used to examine the expression levels of the indicated transposon and genes. The mRNA levels were normalized to the levels of rp49. (B) The same total RNA samples used in (A) were subjected to strand-specific RT-qPCR analysis for RNAs derived from 42AB. The location of amplicon used in the qPCR reaction is as indicated in S10B Fig. (C) The H3K9me3 levels at 42AB (cl1-A and cl1-32), the 5’ region of Burdock (Burdock 5’) and the promoter of HeT-A (HeT-A pro) in ovaries were analyzed by ChIP-qPCR. The H3K9me3 IP signals were first normalized to the histone H3 IP signals (H3K9me3/H3), and then the H3K9me3/H3 values were normalized to the mean of H3K9me3/H3 values obtained for the rp49 loci in EGFP RNAi-1 ovaries, which was set as 1. The location of amplicons used for 42AB is the same as indicated in S10B Fig. The amplicon for Burdock 5’ is located at the position 152–283 of a full-length Burdock insertion, and that for HeT-A pro is located at the position 4094–4240 of the HeT-A{}4795 insertion [37]. In (A-C), data represent the mean of three biological replicates +/- SD. *P < 0.05, **P < 0.01, ***P < 0.001 (Student's t-test). Genotypes of the females are as described in S10 Fig. (TIFF) [file pgen.1009349.s014.tiff]

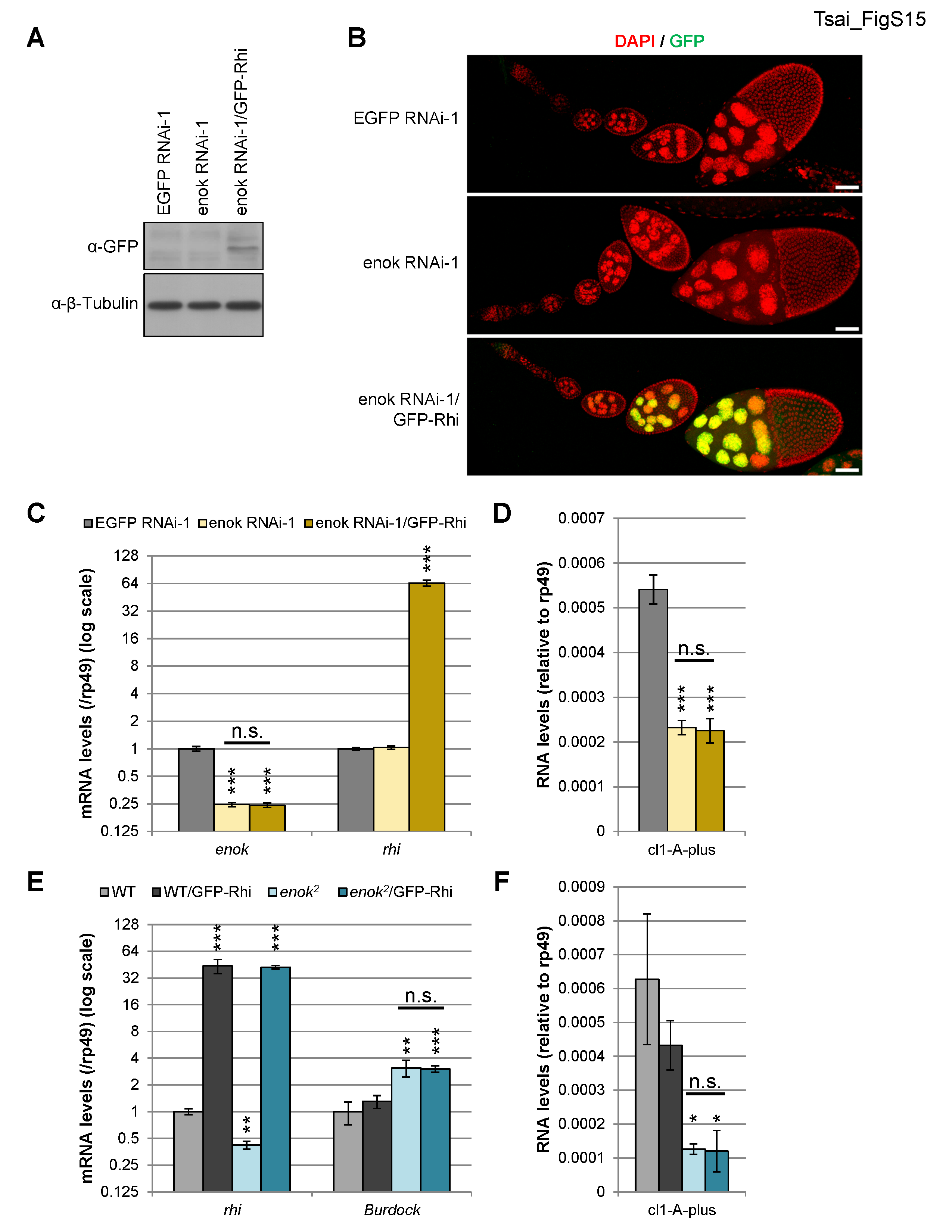

Supplement: S15 Fig — (A) Whole cell extracts were prepared from ovaries and subjected to western blotting. β-tubulin was used as the loading control. (B) Ovarioles containing the germarium and egg chambers up to stage 9 were stained with DAPI. Bars: 50μm. (C) RT-qPCR analysis of ovaries was used to examine the expression levels of the indicated genes. The mRNA levels were normalized to the levels of rp49. (D) The same total RNA samples used in (C) were subjected to strand-specific RT-qPCR analysis for RNAs derived from 42AB. The location of amplicon used in the qPCR reaction is as indicated in S10B Fig. (E) RT-qPCR analysis of ovaries was used to examine the expression levels of the indicated gene or transposon. The mRNA levels were normalized to the levels of rp49. (F) The same total RNA samples used in (E) were subjected to strand-specific RT-qPCR analysis for RNAs derived from 42AB. The location of amplicon used in the qPCR reaction is as indicated in S10B Fig. In (C-F), data represent the mean of three biological replicates +/- SD except for enok2, which represent two biological replicates. *P < 0.05, **P < 0.01, ***P < 0.001 (Student's t-test). n.s.: not significant. Genotypes of the females are as follows: + / CyO; P{w[+mC] = GAL4::VP16-nos.UTR}CG6325MVD1 / P{VALIUM20-EGFP.shRNA.1}attP2 (EGFP RNAi-1); + / CyO; P{w[+mC] = GAL4::VP16-nos.UTR}CG6325MVD1/ P{TRiP.HMS02634}attP2 (enok RNAi-1); +/CyO; P{w[+mC] = GAL4::VP16-nos.UTR}CG6325MVD1, UASp-rhi:GFP /P{TRiP.HMS02634}attP2 (enok RNAi-1/GFP-Rhi); hs-Flp / +; FRTG13 / FRTG13, ovoD1-18 (WT); hs-Flp / +; FRTG13/ FRTG13, ovoD1-18; P{w[+mC] = GAL4::VP16-nos.UTR}CG6325MVD1, UASp-rhi:GFP / + (WT/GFP-Rhi); hs-Flp / +; FRTG13, enok2 / FRTG13, ovoD1-18 (enok2); hs-Flp/ +; FRTG13, enok2/FRTG13, ovoD1-18; P{w[+mC] = GAL4::VP16-nos.UTR}CG6325MVD1, UASp-rhi:GFP/+ (enok2/GFP-Rhi). (TIFF) [file pgen.1009349.s015.tiff]

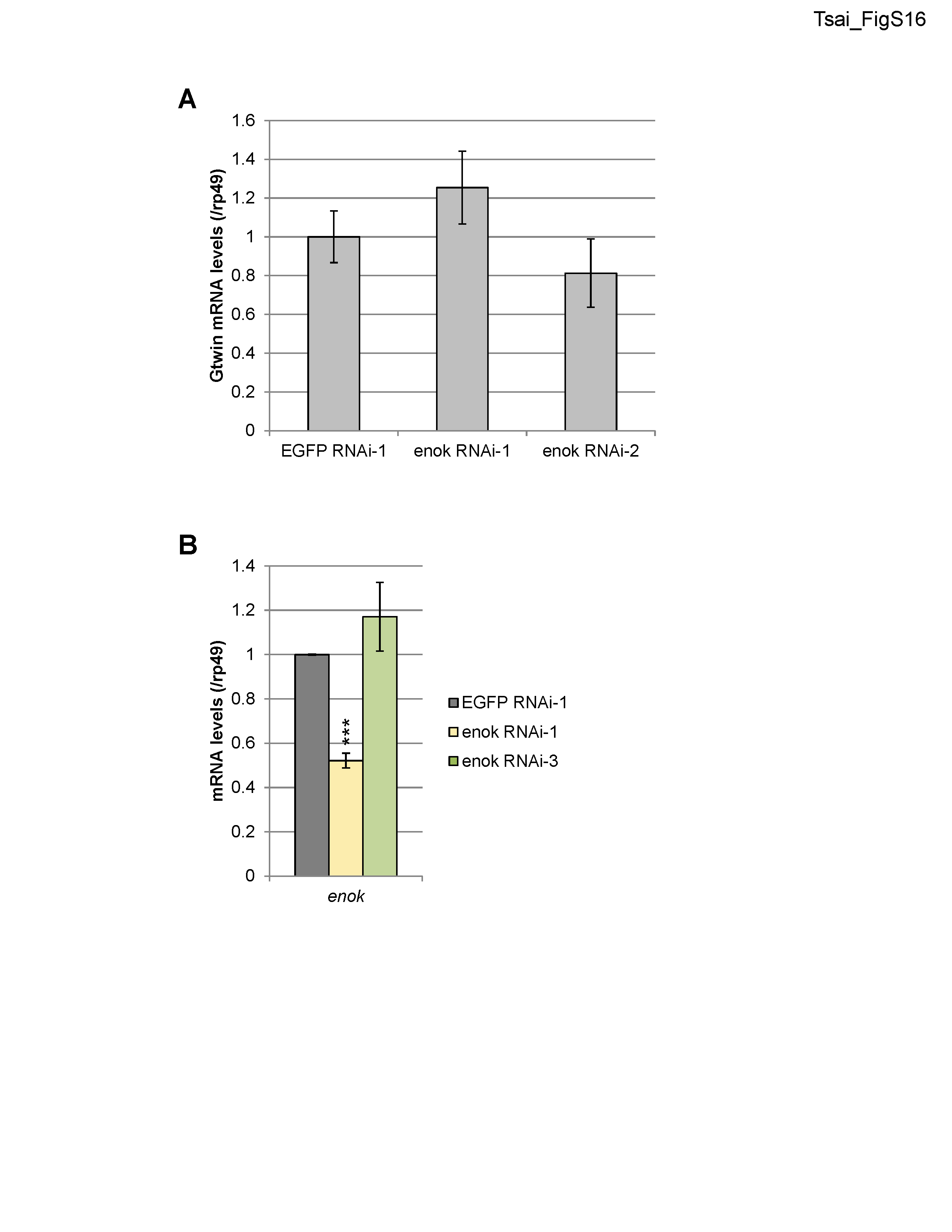

Supplement: S16 Fig — (A) RT-qPCR analysis of ovaries was used to examine the expression levels of Gtwin. The mRNA levels were normalized to the levels of rp49. Genotypes of the females are as follows: tj-GAL4/+; P{VALIUM20-EGFP.shRNA.1}attP2/+ (EGFP RNAi-1); tj-GAL4/+; P{TRiP.HMS02634}attP2/+ (enok RNAi-1); tj-GAL4/+; P{TRiP.HMS02048}attP2/+ (enok RNAi-2). (B) RT-qPCR analysis of ovaries was used to examine the expression levels of enok. The mRNA levels were normalized to the levels of rp49. Genotypes of the females are as follows: P{w[+mC] = UAS-Dcr-2.D}1 / +; P{w[+mC] = GAL4-nos.NGT}40 / +; P{VALIUM20-EGFP.shRNA.1}attP2 / + (EGFP RNAi-1); P{w[+mC] = UAS-Dcr-2.D}1 / +; P{w[+mC] = GAL4-nos.NGT}40 / +; P{TRiP.HMS02634}attP2 / + (enok RNAi-1); P{w[+mC] = UAS-Dcr-2.D}1/ +; P{w[+mC] = GAL4-nos.NGT}40/ +; P{TRiP.HM05195}attP2/+ (enok RNAi-3). In (A-B), Data represent the mean of three biological replicates +/- SD except for EGFP RNAi-1 in (B), which represent two biological replicates. ***P < 0.001 (Student's t-test). (TIFF) [file pgen.1009349.s016.tiff]
