# Supplementary material for: Acetyltransferase Enok regulates transposon silencing and piRNA cluster transcription
Source: PLoS Genet. 2021 Feb 1;17(2):e1009349. doi: 10.1371/journal.pgen.1009349 (PMC7877743; doi:10.1371/journal.pgen.1009349)
Supplement: Attachment — Submitted filename: Tsai et al_Responses to reviewers comments.docx [file pgen.1009349.s024.docx]

July 16th, 2020

Re: PGENETICS-D-20-00022

Dear Drs. Chambeyron and Bickmore,

Please find below our response to the reviewers’ comments, a description of new experiments and analyses added to the manuscript, and changes to the text. We thank you for granting us the time necessary to carry out new experiments and analyses to address the reviewer’s comments. We sincerely thank the reviewers for their insightful comments. The suggested experiments and analyses have greatly helped strengthen the content and conclusions of our manuscript. Please see below a detailed discussion of these points and what we have done to address them.

---------------------------------------------------------

**Reviewer #1**: Tsai and Huang describe a novel function of the Drosophila acetyltransferase Enok in transcription of piRNA precursors and Rhino recruitment to a subset of the germline piRNA clusters. The data presented in the manuscript undoubtedly have a potential interest for the piRNA field, however, the analysis seems to be superficial and not conclusive.

Specific comments

1. Lines 78-81. Information about the presumed function of H3K23ac histone mark in the Introduction is poor and should be enhanced.

The role of H3K23ac is relatively understudied and very little information is known about this histone modification. We have added the following description in the Introduction (Lines 81-83) to enhance the information: “H3K23ac has been suggested to destabilize the interaction between H3K27me3 and the chromodomain of Polycomb (21), and therefore may contribute to transcription activation.”

2. Fig1A – can you define the Y axis (TPM, log2TPM?)

The Y axis shows reads per million (RPMs). We have added this information in the legend.

3. Figs S1, S2 – PCA plots are more appropriate to demonstrate similarity of RNAseq replicates

We thank the reviewer for this advice. We have added the PCA plot in Fig S3, which shows the replicates with the same genotype are grouped closer together as compared with the replicates with different genotypes.

4. Different effects on TAHRE, hobo and rover expression in enok1 and enok2 could be explained by the inter-strain polymorphism in the TE copy number. It should be verified.

As the Reviewer #2 suggested, we have used TEtranscripts (differential analysis using DESeq2) to analyze our RNA-seq data. As a result, we now only included the seven TEs that show significant activation (adjusted p_value <0.05; Table S1) in both *enok* mutants as compared with the WT control in Fig 1C. Actually *TAHRE* is significantly activated in both *enok* mutants as analyzed using TEtranscripts. While we removed *hobo* and *rover* from Fig 1C, we indeed examined the copy number of *rover* in genomic DNA by qPCR using *rp49* as the internal control for normalization. As shown in the chart below, both *enok1* and *enok2* show similar copy numbers of *rover*.

Normalized amount in genomic DNA (/rp49)

|  | enok1 | enok2 |
| --- | --- | --- |
| rover | 1 | 1.04672 |

Primers used for *rover* are:

rover F, 5’-CCTCAAACGTTGGCTCGAAAC-3’;

rover R, 5’-CTTAATTTGGTCTCGTTGTCGTCAG-3’.

5. Lines 124-125 – the conclusion that “Enok is important for proper transposon silencing in the germline” is not quite correct. Specifically, 7 TE families were significantly activated in enok mutants. Some kind of specificity is observed for Enok function and this fact should be discussed.

We thank the reviewer for this suggestion. We have changed this conclusion (Lines 120-121) to “Enok is important for proper silencing of 7 transposon families in the germline.” To address the possible specificity for Enok function, we have analyzed the fold change in transposon family expression in *enok* mutants (Fig S8; y axis, enok1/WT or enok2/WT) and the fold change in antisense piRNAs encoded by the same transposon family (Fig S8; x axis, enok1/WT or enok2/WT) as performed in the previous study for the *rhi* mutant (Klattenhoff et al., 2009, Cell). The results for *enok* mutants (Fig S8) are similar to those for the *rhi* mutant (Fig S10 in Klattenhoff et al., 2009): some of the highly over-expressed transposons (*Burdock* and *HeT-A*) also show large reductions in antisense piRNAs, while expression of *blood* increases 10-12 fold but the total antisense piRNA pool is reduced by only 12%-25%. This similarity between *enok* and *rhi* mutants further supports our hypothesis that Enok may promote piRNA cluster transcription by regulating *rhi* expression and recruitment of Rhi to those clusters. We have added the following description in the Discussion (Lines 389-393) to discuss this specificity observed for Enok function: “The *enok* and *rhi* mutants show similar effects on the fold change in transposon family expression and in antisense piRNAs (Fig S8) (15). However, among the top 24 most highly over-expressed families in *rhi* (15), loss of Enok in the germline specifically activates 7 families. This specificity suggests that these 7 families may be more sensitive to reductions in Rhi recruitment to a subset of piRNA source loci.”

6. Lines 128-137. This paragraph is confusing. Can you explain why transcriptions of sense and antisense genomic strands in a subset of dual-strand clusters are regulated independently.

A previous study has shown that there are multiple transcription start sites (TSS) across several Rhi-dependent clusters including 42AB (Andersen et al., 2017, Nature). Therefore, it is possible that each or groups of those TSS within a cluster are regulated independently. Comparing the bottom left panel of Fig 4B and Fig 6B, we can see the Rhi levels are higher in the region upstream to cl1-32 (Fig 4B) in the “minus” direction than in the region upstream to cl1-32 in the “plus” direction. Thus, upon *enok* knockdown, it is likely that the remaining Rhi levels in the region upstream to cl1-32 in the minus directions (Fig 6B; *enok*_KD) are still sufficient to support transcription, resulting in normal transcript levels at “cl1-32-minus” in the *enok RNAi-1* ovary in Fig 4B (upper left panel; yellow bar).

7. Lines 138-140 – definition of piRNA source loci and the difference between piRNA clusters and piRNA SL should be provided here. The differences in the number of piRNA clusters (140) and piRNA SL (several thousands) are especially confusing and have to be clearly explained.

We apologize for the confusion. We have now added the following sentences in the text (Lines 150-154) to explain the difference between piRNA clusters and piRNA SL: “After the piRNA clusters were first annotated (5), further studies have identified more regions in the fly genome that give rise to piRNAs (14). Therefore, all the genomic regions giving rise to piRNAs were divided into 1 kb bins and defined as piRNA source loci (piRNA-SL). These piRNA-SL includes >91% of 1 kb bins defined by previously annotated piRNA clusters and extends the size of piRNA source regions more than 3-fold (14).”

8. Line 158 – the authors conclude that 20% of the Rhi-dependent loci are Enok-dependent (ED). Annotation of ED piRNA loci has to be performed in order to find similarities between these loci and to explain a possible mechanism of Enok targets selectivity (see also #15 comment).

The annotation of ED-SL and EI-SL is now provided in the newly added Table S3. ED-SL are located in both the telomeric and centromeric piRNA clusters/source loci. We have also colored the 1kb bins in 42AB and cluster5 (mostly ED-SL) and cluster38C1 (EI-SL) in Figure S6A to show the Enok-dependency of these representative piRNA clusters.

9. Lines 177-178. Western blotting analysis is needed here to confirm reduction in the Rhi protein levels in the enok mutant ovaries.

Because the anti-Rhi antibody does not work for Western blotting (Mohn et al., 2014, Cell), the only way to examine the Rhi protein levels in the *enok* mutant ovaries by WB is to generate *enok* mutant germline clones with the *GFP-rhi* transgene used in Figure S12C-E. We have tried to generate the *hsFLP/+; FRTG13/ovoD, FRTG13; GFP-rhi/+* (WT control) and *hsFLP/+; enok^1^, FRTG13/ovoD, FRTG13; GFP-rhi/+* (*enok* mutant) flies. These females can only be generated through 4 crosses in a row, so we only obtained limited numbers of females. Also, in the final cross, only half of the females would carry the *GFP-rhi* transgene. In addition, germline clone ovaries (including the WT control) always develop poorly (roughly ⅕ of the normal ovary). We dissected as many ovaries as possible (~30 females in each group) and performed WB. Unfortunately, as the GFP-Rhi levels are quite low for WB (Fig S12C), we were not able to detect its level even in the WT control germline clone ovaries. In the text, we emphasized on the regulation of transcript levels of *rhi* by Enok (Fig 3 and S7A), and the immunostaining of Rhi was only used as supportive data (Fig S7C). Furthermore, we have shown that overexpression of *rhi* cannot rescue the defective RNA levels transcribed from 42AB in the *enok^2^* mutant germline clone ovaries (Fig S15E-F). These results suggest that Enok is indeed important for the recruitment of Rhi to a subset of piRNA source loci even when Rhi protein levels are high.

10. Line 184. Specificity of a-Enok antibodies has to be verified by WB using ovary extracts from wild type and knockdown strains.

Specificity of the anti-Enok antibody used in our ChIP assay was verified in the previous study (Huang et al., 2014, G&D) by WB (cell extracts from control and knockdown; 0-3 hr embryo extracts from wild-type and mutant germline clone females) and ChIP-qPCR (from control and knockdown cells). In this study, we have also included a ChIP-seq sample using another independently raised anti-Enok antibody (#2) to show that these two antibodies (#1 and #2) resulted in very similar ChIP-seq profiles (Fig S9). This result further supports the specificity of our Enok ChIP-seq datasets.

11. a-H3K23ac ChIP data (Fig S5b) are essential and would be better moved in the main figure.

We thank the reviewer for this suggestion. We have now moved this figure to Figure 3E.

12. Lines 251-253. A plausible explanation should be proposed to explain such various effects of enok mutations and knockdowns on expression of different targets, namely, 42AB and Rhi. In enok_KD, H3K23ac staining (Fig S8D) as well as H3K23ac enrichment at Rhi promoter (Fig. S5B) are significantly decreased. In lines 201-202 the authors state that “Enok may promote the expression of rhi and mael by acetylating H3K23”. Therefore, the situation is controversial, because enok_KD affects H3K23ac levels at Rhi promoter but does not affect Rhi levels. Can you clarify this? At least, H3K23ac enrichment at Rhi promoter has to be tested upon “mild” enok_KD.

We apologize for the confusion. Indeed, we showed that the H3K23ac levels at the 5’ end of *rhi* were reduced ~30% upon *enok* knockdown (Fig 3E). Consistently, the mRNA levels of *rhi* were also reduced ~30% upon *enok* knockdown (Fig S10A and S14A; yellow bars). These results confirm that the reductions of H3K23ac levels and *rhi* mRNA levels in *enok*_KD roughly match each other. However, a 30% reduction in mRNA levels is considered subtle (better than a heterozygote) and may not affect the protein level if the wild-type mRNA level is originally a little in excess under the normal condition. Therefore, we examined the protein level of Rhi in *enok*_KD and found that the Rhi protein levels indeed remained unaffected when the *rhi* mRNA levels were reduced 30% (Fig S12B-S12E). This result also suggests that the *rhi* mRNA levels may be a little in excess as compared with its protein levels under normal condition.

13. The authors statement that “knocking down enok in the germline can decrease levels of RNAs from 42AB without affecting the global Rhi levels (Fig S8B-S8E)” is needed to be confirmed by more convincing experiments. Rhi-GFP protein levels have to be estimated more precisely using titration of the ovary extract and quantification. Loading control panel (Fig S8C) is overexposed that does not allow quantification. RT-qPCR of the Rhi RNA levels is highly recommended to back up the Rhi protein analysis in the ovaries of “mild” enok_KD.

We have replaced the loading control panel (now Fig S12C) with a less-exposed image. Since WB is not ideal for quantification, we used the fluorescence microscopy data for quantification. As shown in Figure S12E, the H3K23ac and GFP (Rhi) signals were normalized to the DAPI signals, and the n-numbers of each group are >=13. The quantification results show that the GFP-Rhi levels are similar in the control and the *enok* knockdown ovaries.

As to clarify the data obtained under “mild” or “strong” *enok*_KD condition, only the data shown in Figure 4 were obtained using “mild” *enok*_KD. All other *enok* knockdown data were collected under the “strong” *enok*_KD condition. Therefore, Figure 4A shows the *rhi* mRNA levels in the ovaries of “mild” *enok*_KD (yellow bars). However, Figures S12B-S12E were performed in the ovaries of “strong” *enok*_KD, and the *rhi* mRNA levels are shown in Figures S10A and S14A (yellow bars).
We apologize for the confusion.

14. Along the text, the expression “We have previously demonstrated…” should be replaced by ”it was previously demonstrated” because only one of the co-authors was involved in the mentioned studies before moving to another lab.

We apologize for the error. We used this expression because the corresponding author of this current study is the first author of that previous study. We have now replaced this expression with the suggested one along the text (for example, Lines 217 and 226).

15. The authors discuss the regulation of piRNA-precursors transcription by Enok-mediated Rhi recruitment to the specific group of the germline piRNA clusters, however, no attempts were done to annotate the clusters related to this group. Recently, the specificity of the regulation of particular subsets of piRNA clusters, such as telomeric regions and euchromatic transposons, was demonstrated (Radion et al 2018, Kordyukova et al 2020). These data would be helpful for annotating Enok targets.

We thank the reviewer for the suggestion. Radion et al 2018 demonstrated that the recruitment of Rhi to telomeric regions and 42AB, respectively, showed different dependencies on piRNAs. Kordyukova et al 2020 showed that Ccr4-Not specifically degrades subtelomeric transcripts without affecting nontelomeric piRNA clusters (including 42AB). However, as shown in Table S3, Enok-dependent piRNA source loci (ED-SL) are located in both the telomeric and nontelomeric piRNA clusters. Therefore, the specificity of regulation of a subset of piRNA clusters/source loci by Enok may not be determined merely by telomeric properties. These results suggest that there may be other yet unknown factors/mechanisms that target and regulate the function of Enok to facilitate Rhi recruitment and that, while interesting, sorting out this complexity will require extensive further analysis.

**Reviewer #2**: The last author of the present manuscript is also first author of a 2014 publication, which shows that the histone acetyltransferase Enok promotes expression of Spire and Maelstrom (Mael). Mael is involved in piRNA-mediated silencing of transposable elements (TEs) in Drosophila germline cells (Sienski et al. 2012). In the present manuscript, the authors were interested in a possible role of Enok in TE silencing. They examined TE transcription, production of small RNAs by piRNA clusters and by genome-wide 1-kb bins, they studied gene transcription levels, especially for genes involved in the piRNA pathway, as well as Enok, Rhino, and RNA polII occupancy on DNA, H3K23 acetylation, and Rhino, Mael and Piwi protein expression. These studies were done in Enok mutant germline clones, Enok-RNAi knock-down (KD) and control conditions.

Three transcriptome-wide RNAi screens identified genes, which are involved in the piRNA pathway in Drosophila, one screen for the germline and two for ovarian somatic cells (ref. 23, 25, 26 in the present manuscript). None of these screens did identify Enok as a possible candidate to be involved in the piRNA pathway and TE silencing. This discrepancy with the data presented here is discussed in the present manuscript (Lines 351-362). Still this discussion is based on unpublished data (Lines 356-357).

We have performed an RT-qPCR assay in ovaries with control or *enok* RNAi constructs driven by the nos-GAL4 driver in the presence of a *Dcr2* transgene (the same driver line used in the original ref. 23). The new data is now shown in Figure S16B. First, the knockdown strength of *enok RNAi-1* driven by nos-GAL4 is weaker than that driven by MTD-GAL4 as used in this study (comparing Fig S16B and Fig S10A). Second, the knockdown strength of *enok RNAi-1* (short hairpin RNA) is stronger than that of *enok RNAi-3* (long hairpin RNA). Therefore, this result supports our discussion (Lines 401-402): “The efficiency of knocking down *enok* by long hairpin RNAs is lower than by short hairpin RNAs in the germline, even in the presence of additional Dicer-2.”

The manuscript is well written and the authors present here large amounts of data, obtained by RNA-seq, small-RNA-seq, ChIP-seq, ChIP-qPCR, RT-qPCR, Immunostaining and Western-blot, but unfortunately, the data as presented cannot be considered as conclusive. All statistical tests to check differential expression or IP seem to be simple T-Tests, for only 3 replicates of each condition, only 3 values being used for each T-test. Moreover, the correlation coefficients between many replicates are quite low (see comments). DESEQ or edgeR analyses will thus be required for all analyses of differential expression of transcripts (RNA-seq), small RNAs (small-RNA-seq), or of ChIP-Seq data. These more adequate statistical tools use the original non-normalized counts and are based on variance within the samples and between the samples. Simple T-tests are not sufficient to draw the right conclusions here. For gene expression, such analyses shall be done on all mRNAs, not only on genes of the piRNA pathway to be statistically consistent. The use of Wilcoxon Rank sum test for many of the presented data is not adequate in its implementation, allowing doubts on the drawn conclusions.

Please see our detailed responses to each comment below.

Concerning data obtained by qPCR, the primers present major defaults, several of them being non-specific, matching several times in the Drosophila genome presenting only 1 or 2 mismatches with off-target regions, other primers, supposed to be specific of one single locus, are so small, 11-13 nucleotides, that they match without mismatch up to 25 different loci in the Drosophila genome. The corresponding qPCR results, notably for piRNA cluster expression, cannot be conclusive (see also detailed comments).

Please see our detailed responses to the comment below.

In conclusion, in spite of the huge amount of data and possibly interesting results that may emerge if statistics are done correctly, the manuscript, at this point, is not suitable for publication. It needs major revision with profound changes concerning all statistics and primer design as suggested in my detailed comments before re-submission.

Major comments:

The authors never indicate which are the "Enok-dependent piRNA source loci". They correspond to specific 1-kb bins, but the authors do not indicate whether they are located in piRNA clusters, in the 42AB piRNA cluster, which is analysed in detail, or whether they are in totally different genomic regions, eventually even dispersed all over the genome. This important information remains obscure.

We thank the reviewer for this suggestion. The annotation of ED-SL and EI-SL is now provided in the newly added Table S3. ED-SL are located in both the telomeric and centromeric piRNA clusters/source loci. We have also colored the 1kb bins in 42AB and cluster5 (mostly ED-SL) and cluster38C1 (EI-SL) in Figure S6A to show the Enok-dependency of these representative piRNA clusters.

There is no link evidenced between piRNA cluster down regulation and the suggested up-regulation of 12 TE families. Actually, the authors do not show whether small RNAs complementary to these 12 TE families are down-regulated.

As the Reviewer suggested, we have used TEtranscripts (differential analysis using DESeq2) to analyze our RNA-seq data. As a result, we now only included the 7 TEs that show significant activation (adjusted p_value <0.05; Table S1) in both *enok* mutants as compared with the WT control in Fig 1C. We have also analyzed the fold change in transposon family expression in *enok* mutants (Fig S8; y axis, enok1/WT or enok2/WT) and the fold change in antisense piRNAs complementary to the same transposon family (Fig S8; x axis, enok1/WT or enok2/WT) as performed in the previous study for the *rhi* mutant (Klattenhoff et al., 2009, Cell). The results for *enok* mutants (Fig S8) are similar to those for the *rhi* mutant (Fig S10 in Klattenhoff et al., 2009): some of the highly over-expressed transposons (*Burdock* and *HeT-A*) also show large reductions in antisense piRNAs, while expression of *blood* increases 10-12 fold but the total antisense piRNA pool is reduced by only 12%-25%. This similarity between *enok* and *rhi* mutants further supports our hypothesis that Enok may promote piRNA cluster transcription by regulating *rhi* expression and recruitment of Rhi to those clusters.

Primer design (Lines 515-561):

Several primers that were used to detect piRNA cluster or gene transcription are not specific (for example cl1-A-plus, cl1-32-plus, enok F). Indeed they map not only the locus aimed to be amplified but also elsewhere in the genome with only 1 or 2 internal or 5'-end mismatches (Flybase Blast, E=1000, no low complexity filter). Their 3'-end is not specific to the locus aimed to be amplified (see specific primer design for repeated sequences and its requirements in de La Roche Saint Andre C. and Bregliano J.C., Genetics 1998). Other primers are much too short to be specific (cl2-A-plus - 11 nucleotides, 25 matching loci in Release 6 genome; cl2-A-minus - 13 nucleotides, 11 matching loci in Release 6 genome) or contain palindromic sequences at their 3'-end (cl1-32-minus), presenting a risk of auto-amplification.

The cl1-A-plus, cl1-32-plus, cl2-A-plus, cl2-A-minus, and cl1-32-minus primers were used for the strand-specific RT reaction. Indeed, they may prime transcripts from more than one place in the genome. However, the primer sets used for the following qPCR reaction are specific for the indicated sites. The sequences of all primers used for strand-specific RT or qPCR reactions for cl1-A, cl1-32 and cl2-A were reported in the previous study (Klattenhoff et al., 2009, Cell). Also, below are the melting curve analysis results of cl1-A, cl1-32, and cl2-A, which show that all these 3 primer sets amplify only one specific fragment.

cl1-A:

cl1-32:

cl2-A:

As for enok F, indeed, it maps to another two places in the genome that are far away from the target site. However, the enok R primer is specific. Therefore, within the 45-sec extension time, only one specific fragment can be amplified, as shown in the melting curve analysis plot below:

For primers destined to amplify TEs, it would be suitable to state whether they target specifically putative functional TEs or whether they may also amplify ancestral TE copies, which may be present in piRNA clusters. At least, it would be necessary to know whether the primers for TEs that seem to be de-silenced in enok mutant or KD conditions detect sequences within piRNA clusters, notably the 42AB cluster.

We thank the reviewer for this suggestion. We have added the following statement at the end of the Primers section in Materials and Methods (Line 620): “All amplicons for *Burdock*, *Blood* or *HeT-A* are not located within 42AB.”

In several Figures appear p-values that have been calculated by a Wilcoxon Rank sum test. These p-values are extremely low and do not seem to be in adequacy with the corresponding box-plots. The Methods section gives details on how this test was implemented (Lines 446-452). It appears that the Wilcoxon Rank sum test has been done on means from 3 replicates for each 1-kb bin ("average numbers of reads from 3 independent replicates"). The authors have 3 replicates for each condition and they want to test whether medians of control samples are significantly different from the medians of mutant or knock-down (KD) samples. To do this a Kruskal-Wallis test will be needed. The results should then appear in a matrix and show no difference between replicates of the same condition, and significant difference between each control sample and each mutant or KD sample.

We thank the reviewer for pointing out this issue. We have now followed the procedure described in the original study using this 1-kb bin analysis (Mohn et al., 2014, Cell) to pool the raw data from 3 replicates before normalization to library depth and mappability. The resulting processed data were then suitable for the Wilcoxon Rank sum test. All the Figures showing results of 1-kb bin analysis were updated with the new processed data, and all of them support our original hypothesis/conclusions.

Lines 109-111: "Given the high degree of correlation between each independent replicate in our next generation sequencing (NGS) data sets (Fig S1 and S2), ..."

"High degree of correlation" may be true for RNA-seq data (Fig. S2A), but not for the other data, where, the correlation coefficients between many replicates are quite low, 2 out of 3 coefficients being <0.90 for all small-RNA-seq replicates (Figure S2B), 1 or 2 of 3 <0.90 for Enok ChIP-seq replicates (Figure S2C), and 1 out of 4 coefficients being <0.80 for replicates from RNA Pol II ChIP-seq (Figure S2D).

Indeed, the correlation coefficients between some of our small RNA-seq or ChIP-seq (IP) replicates are between 0.85 and 0.90. Also, two coefficients between the ChIP Input replicates are between 0.75 and 0.80. However, Chan YH, 2003, Singapore Med J (44(12):614-619) defined the strength of linear relationship with a coefficient value more than 0.80 as very strong, and that with a coefficient value between 0.60 and 0.80 as moderately strong. In addition, Mukaka MM, 2012, Malawi Med J (24(3):69-71) defined the correlation with a coefficient value between 0.70 and 0.90 as high correlation (0.90-1.00 as very high). Therefore, the correlation coefficients between our NGS replicates should be able to support that the degree of correlation between them is high.

Lines 112-118:

I assume that Figure 1A shows all mapping reads, not only genome-unique mappers. Still this is not specified. Multiple-mappers may originate from other loci than the ones shown in the figure. Thus why do the authors present these specific loci for Burdock and HeT-A ? It is not even sure that these insertions exist in the genomic context used here.

We have specified that we used all mappers in RNA-seq analysis for transposon families in the Material and Method section (Line 474-475). Since it is impossible to use unique mappers for transposons and we cannot distinguish where the multiple mappers originate from (as the reviewer pointed out), we showed representative copies of transposons in Figure 1A. We showed *Burdock{}783* because it is next to two protein-coding genes, and therefore the genome browser view also demonstrates that the expression levels of these two genes were not affected. In the right panel, we presented several copies of *HeT-A* to show the patterns of different copies of *HeT-A*. In addition to showing the representative copies of transposons, we also performed systematic and genome-wide analysis on transposon expression as shown in Figures 1B,1C and Table S1.

One might assume that the data shown in Figure 1B and 1C correspond to RNA-seq data, as well as the data in "Table S1_TPMs of transposons.xlsx" but this is not specified. Moreover, Figures 1B and 1C show different results than Table S1.

Example: mean TPM from Table S1 for Burdock: wt - 1.1; enok1 - 21.6; enok2 - 16.9

Figure 1B,C show the following values for Burdock: wt - around 8; enok1 - around 100; enok2 - around 100

The same discrepancies are observed for other transposable elements in Figure 1B and 1C. In fact, the data in Figures 1B and 1C seem to be related to Table S1, but the scale is clearly erroneous (100 does clearly not correspond to 1 TPM, 101 does not correspond to 10 TPM etc.).

We indeed indicated that the data are obtained from RNA-seq in the legends of Fig 1C and Table S1. We have now added the following description in the legend of Fig 1B: “The levels of mRNAs, non-coding RNAs and transposons in *enok* mutant germline clone ovaries versus the WT control detected by RNA-seq are plotted in a log scale.”

We have checked the values shown in Fig 1C and they indeed matched the TPM values shown in Table S1. As for Fig 1B, it shows the “normalized number of reads” generated by piPipes (please also see the Material and Method section) but not TPM.

Table S1 ("Table S1_TPMs of transposons.xlsx"):

What do values like "9,6119E-115" (FBgn0001100_G-element) and "1,4906E-188" (FBgn0020425_Helena) mean, in a table supposed to show TPM (transcripts per million) ? Moreover, it is not clear what "TPM" means. How were these values calculated?

The concept of TPM was first proposed in 2012 (Wagner et al., 2012, Theory Biosci), and it is modified from RPKM to make the average relative molar RNA concentration for each set of transcripts a constant. In this study, we used piPipes (Han et al., 2015, Bioinformatics) to calculate the TPM for each transposon family (please also see the Material and Method section).

Table S2 ("Table S2_abundance of piRNAs mapping to piRNA clusters.xlsx"):

What values are given in this table, RPKM, RPM, normalized to all genome-unique reads, of which size ? The title of the table and the title of the sheet it contains suggest that these are genome-unique piRNA counts, but this is not specified in the legend or in the table. And again, as no Piwi-IP has been done to sort out piRNAs, what is the criterion to name these reads "piRNAs" ?

Table S2 shows the normalized piRNA reads assigned to each piRNA cluster as calculated by piPipes (normalized to miRNAs) (Han et al., 2015, Bioinformatics), and these normalized piRNA reads were defined as the “abundance” of piRNAs in piPipes. We have added the following sentence in the legend of Table S2 to improve the clarity: “The unique reads mapping to each piRNA cluster were normalized to miRNAs and defined as the abundance of piRNAs.”

We followed the recommendation by the authors of piPipes to define the length of piRNAs, which is 23–29 nt, when using piPipes to analyze our small RNA-seq data.

Lines 138-139:

It seems that the "piRNA source loci" correspond to 1-kb bins. How have these 1-kb bins been selected ? Are they contained in the 142 piRNA clusters identified by Brennecke et al., 2007 ? Even if this has been described in ref. 13, it is not clear to the reader whether the cutoff value of piRNA counts per bin was the same as in ref. 13. Indeed, this cutoff value should depend on the sequencing depth of each sample. Is this the case ? What about p-values to test differential production of small RNAs for single bins. It seems they were not considered here. Again, DESEQ analysis would be suitable. If the used cutoff value of piRNA counts per bin is too low, or variance too high, 1.5-fold changes may not be significant.

After the piRNA clusters were first annotated (Brennecke et al., 2007, Cell), further studies have identified more regions in the fly genome that give rise to piRNAs (Mohn et al., 2014, Cell). Therefore, all the genomic regions giving rise to piRNAs were divided into 1 kb bins and defined as piRNA source loci (piRNA-SL). These piRNA-SL includes >91% of 1 kb bins defined by previously annotated piRNA clusters and extends the size of piRNA source regions more than 3-fold (Mohn et al., 2014, Cell).

Mohn et al., 2014 indeed applied some cutoff criteria to define the piRNA-SL (1 kb bins), including giving rise to at least ten piRNAs in the control sample, more than 10% uniquely mappable sequence, and not overlapping with assembly gaps. However, they did not apply any cutoff when using the 1 kb bins to analyze their NGS data. They actually added a pseudocount of 0.1 to the value of reads in each bin after normalization to library depth and mappability, in order to solve the problem that some bins would have zero reads. We instead excluded the bins with zero reads from the analysis. Therefore, we performed this 1 kb bin analysis with a more stringent criterion as compared with the original study (Mohn et al., 2014, Cell).

We have performed DESeq2 analysis on piRNA reads mapping to each piRNA cluster. The results are now included in Table S2 (the second worksheet). We have also updated Fig S4 with the results from DESeq2 analysis.

Lines 157-159: "Thus, we concluded that Enok may be important for piRNA production from ~20% of the Rhi-dependent loci."

This conclusion concerns 1-kb bins and nothing indicates whether these "20% of the Rhi-dependent loci" correspond to real "loci", for example piRNA clusters or whether they are dispersed over the genome. This would be essential to examine.

We thank the reviewer for the suggestion. The annotation of ED-SL and EI-SL is now provided in the newly added Table S3. ED-SL are located in both the telomeric and centromeric piRNA clusters/source loci. We have also colored the 1kb bins in 42AB and cluster5 (mostly ED-SL) and cluster38C1 (EI-SL) in Figure S6A to show the Enok-dependency of these representative piRNA clusters.

Lines 189-190: "Therefore, our results suggest that Enok may contribute to proper piRNA production partly by binding to rhi, mael and shu and facilitating their transcription."

The presented data do not clearly support this conclusion. It seems over-stated. Again DESEQ analyses on RNA-seq, small-RNA-seq and ChIP-seq might help to check whether the differentials are significant. These analyses should be done on all genes, not only on pre-selected genes known to be involved in piRNA biogenesis.

We thank the reviewer for the suggestion. We have performed DESeq2 analyses on RNA-seq, small RNA-seq and ChIP-seq data sets. We defined significant changes as changes with an adjusted p_value<0.05. The results from TEtranscripts (an analysis package using DESeq2 for differential analysis) on RNA-seq are now included in Table S1 to show that 7 transposon families were significantly activated in both *enok* mutant germline clone ovaries. Also, the results from DESeq2 analysis on RNA-seq reads uniquely mapping to piRNA clusters are now shown in Table S5 to demonstrate that transcript levels from 6 piRNA clusters were significantly reduced in both *enok* mutant ovaries. The results from DESeq2 analysis on small RNA-seq are now included in Table S2, and Fig S4 is updated with the DESeq2 results. The results from DESeq2 analysis on H3K23ac ChIP-seq are now shown in Table S6 to show that the H3K23ac levels at piRNA clusters or transposons were not dramatically reduced (log2FoldChange>1) upon *enok* knockdown.

As the RNA-seq data sets were retrieved from the previous study (Huang et al., 2014, G&D), the results of differential analysis on all genes using edgeR were shown in that previous study. Therefore, we only showed pre-selected genes known to be involved in piRNA biogenesis in this current study.

Lines 205-206: "Since rhi is severely down-regulated in enok mutant ovaries, ..."

This seems over-stated. Quantitative analyses are missing at this stage. Corresponding Western-blots are presented later in Figure S8 and their results are not concluding "severe down-regulation".

We apologize for the confusion. The original Figure S8 (now Fig S12) showed data obtained from *enok* knockdown ovaries, but not from *enok* mutant germline clone ovaries. Indeed, the mRNA levels of *rhi* were only reduced mildly (30%) upon *enok* knockdown (Fig S10A; enok RNAi-1). However, as shown in Figure 3A, the mRNA levels of *rhi* were reduced 75% in *enok* mutant ovaries as compared with the WT control, and this reduction in *rhi* expression level is significant as analyzed by edgeR (Table S4; FDR<0.01). Therefore, we indeed showed quantitative analysis supporting that mRNA levels of the *rhi* gene were severely down-regulated in *enok* mutant ovaries.

Lines 263-264: "This result suggests that Enok has a role in promoting transcription of a subset of dual-strand clusters including 42AB."

If the authors have arguments that "a subset of dual-strand clusters" is affected, why do they present only one dual-strand cluster ?

Since 42AB is the major piRNA cluster functioning in the germline, we presented it as a representative cluster. In addition, we have performed DESeq2 analysis on RNA-seq reads uniquely mapping to piRNA clusters according to Reviewer#3’s suggestion. The results are now included in Table S5, and show that the transcript levels from 6 dual-strand clusters (highlighted in purple) were significantly reduced in both *enok* mutant ovaries as compared with the WT control. These results also support that Enok has a role in promoting transcription of a subset of dual-strand clusters. Moreover, we showed that Pol II occupancies at ED-SL were significantly reduced upon *enok* knockdown, while those at EI-SL were unaffected (Fig 5B). As shown in Table S3, many ED-SL are located in dual-strand clusters, including 42AB, cluster5, 13, 29, 30 and 45. Therefore, the 1kb bin analysis (Fig 5B; covering 835 ED-SL and 2912 EI-SL) further suggests that Enok may facilitate transcription of a subset of dual-strand piRNA clusters/source loci.

Lines 266-267: "Consistent with our small RNA-seq results (Fig 2C), Pol II occupancies at ED-SL were significantly reduced upon enok knockdown, while those at EI-SL were unaffected (Fig 5B)."

In addition to solving the problem of implementation of the Wilcoxon Rank sum test (see above), the authors should show data indicating which 1-kb bins have differential Pol II occupancies and whether they are located in piRNA clusters or grouped in other genomic loci.

As mentioned above, we have solved the problem of implementation of the Wilcoxon Rank sum test. The Wilcoxon Rank sum test showed that Pol II occupancies at ED-SL were reduced in *enok* knockdown ovaries as compared with the control knockdown (Fig 5B). The annotation of ED-SL is now provided in the newly added Table S3, with the information of which piRNA cluster they are located in.

Lines 323-327:

The authors propose a model of transposon regulation by Enok in Figure 7D. Unfortunately, in spite of the large quantity of data analysed here, it remains unclear how Enok "regulates the recruitment of Rhi to enhance transcription at piRNA clusters" as concluded by the authors. Moreover, the authors focus on the 42AB piRNA cluster but they do not state whether the transposable elements which seem to be de-silenced according to the presented data (Figure 1C) have related copies being present in the 42AB cluster and whether these copies lead to production of homologous regulating piRNAs in wt conditions.

In this study, we identified Enok as a novel regulatory factor in the germline piRNA pathway, functioning upstream to the recruitment of Rhi to some piRNA clusters/source loci. We showed evidence supporting that Enok regulates transcription at piRNA clusters/source loci by promoting *rhi* expression and by facilitating Rhi recruitment. As for the detailed mechanism by which Enok regulates Rhi recruitment, we speculated in the Discussion section: “As Rhi is highly enriched across the entire 42AB cluster (14) and no Enok peaks were detected within 42AB (Fig 5A), Enok is unlikely to regulate the Rhi occupancy at 42AB by directly recruiting it.”(Lines 426-428) “Interestingly, the HAT activity of Enok is critical for transcription of 42AB even when *rhi* is overexpressed (Fig S15E, S15F). Therefore, it is possible that Enok may play a role in acetylating some factors that are required for Rhi recruitment, or it may have an indirect role in Rhi recruitment by promoting expression of other genes with yet unidentified functions in the piRNA pathway.”(Lines 429-433) However, sorting out these yet unknown factors that are acetylated or regulated by Enok will require extensive further analysis.

As for the relationship between 42AB and the 7 transposon families that are activated in *enok* mutants (Fig 1C), 42AB contains copies of *GATE* and *HMS-Beagle* and these copies indeed lead to production of piRNAs in WT ovaries. In addition, ED-SL contain copies of *Burdock*, *blood*, *TAHRE*, *GATE*, *HMS-Beagle* and *Bari1*, and these copies also lead to production of piRNAs in WT ovaries. As Pol II occupancies at ED-SL were reduced in *enok* knockdown ovaries as compared with the control knockdown (Fig 5B), this result also supports the link between the regulation of piRNA source loci transcription by Enok and the activation of transposons in *enok* mutants. We did not find piRNAs produced from ED-SL that map to *HeT-A*, because only the unique mappers were used in the 1kb bin analysis. However, levels of antisense piRNAs (all mappers) mapping to *HeT-A* were reduced 60% in the two *enok* mutant ovaries (Fig S8). Taken together, these results support that down-regulation of transcription at Enok-dependent piRNA clusters/source loci in *enok* mutants leads to activation of the 7 transposons shown in Figure 1C.

Lines 358-359: "Czech et al. indeed showed that knocking down enok weakly activated the blood and Burdock transposons (less than 2-fold)."

I didn't find this information about fold-change in the cited publication (ref. 23). The corresponding z-scores in this publication were -0,505 and -0,740 respectively, thus non-significant.

We apologize for this error. We have now changed the statement as follows (Lines 402-405): “Czech et al. indeed showed that knocking down *enok* weakly activated the *blood* and *Burdock* transposons (z-scores of -0.5 and -0.74, respectively). However, this activation effect did not reach the threshold (z-score of -1.5 or lower) applied in the screen.”

Discussion:

Regarding the statistical analyses, which are not adequate, the discussion seems overstated and has clearly to be revised, once the data will be re-analysed using the adequate statistical tools and methods.

As detailed in our responses above, we have re-analyzed our data according to the reviewer’s suggestions. The results support our original hypothesis/statements. We have also made minor modifications in the Discussion according to our new results.

Minor comments:

There are not only "truncated transposons" (page 8 line 46) in Drosophila piRNA clusters, they may also contain full-length recent TEs. Please cite Zanni et al., 2013 here for the composition of a typical piRNA cluster.

We have modified the description as (Lines 44-47): “In *Drosophila*, the piRNA pathway starts from transcription of the 142 piRNA clusters, usually ranging from 50 to a few hundred kilobases and containing multiple copies of truncated or full-length transposons, which produces long piRNA precursors (5-7).” Zanni et al., 2013 has been added as Ref 7.

The Piwi protein is not involved in PTGS, only in TGS. Thus the statement "Guided by complementary piRNAs, the Piwi-piRNA complex can mediate degradation of transposon transcripts' (Lines 51-52) is erroneous. The Piwi-piRNA complex enters the nucleus and induces TGS. What is true is that other PIWI-clade proteins "can mediate degradation of transposon transcripts" but not Piwi.

We apologize for the error. We have modified this statement as follows (Lines 51-54): “Guided by complementary piRNAs, Ago3 and Aub can mediate degradation of transposon transcripts, and the Piwi-piRNA complex can also direct heterochromatin formation at the loci of transposons and piRNA clusters by recruiting epigenetic factors (10-13), resulting in effective repression of transposons both transcriptionally and post-transcriptionally.”

Lines 57-59:

The authors state " Transcription of the uni-strand clusters is proposed to be similar to the canonical transcription of protein-coding genes, as they contain clear promoter structures with enriched H3K4me2 and peaks of RNA polymerase II (Pol II) (13)."

I didn't find this information in the cited reference 13. Please verify.

We have verified that Ref.13 contains the following description: “piRNA profiles and steady-state RNA levels confirmed the dual-strand nature of 42AB, while 20A was transcribed exclusively on one strand. Overall, cluster 20A resembles a genic transcription unit and therefore described mouse piRNA clusters (Li et al., 2013): cluster 20A exhibited a defined Pol II recruitment site (transcription start site; TSS; Figure 1C, red arrowhead), enrichment of H3K4me2 at the TSS and an emerging capped transcript.”

Lines 89-91:

"Therefore, Enok contributes to proper transposon silencing in the germline both by activating canonical transcription of rhi and by promoting noncanonical transcription of piRNA clusters."

This conclusion is clearly overstated since non-canonical transcription is not evidenced by the data presented here.

We have modified this conclusion as (Lines 91-92): “Therefore, Enok contributes to proper transposon silencing in the germline by promoting transcription of *rhi* and piRNA clusters.”

Figure 1A:

Which is the unit for the y-axis in Figure 1A, RPKM (reads per kb per million), "TPM" as in Table S1 ? Normalized to genome-mapping reads, all reads, else ? As annotated in Release 6, the gene CG30345 overlaps Burdock in its 3'-UTR. This is not shown in the figure.

The Y axis shows reads per million (RPMs) (normalized to million genome-mapping reads). We have added this information in the legend. Indeed, in the RefSeq Genes track (dm3; Release 5), the 3’-UTR of CG30345 overlaps *Burdock*. However, Figure 1A shows the FlyBase Genes track (dm3), so the 3’-UTR of CG30345 does not overlap *Burdock*.

Line 130: "Among the 138 piRNA clusters in our analysis ..."

Why did the authors analyzed only 138 among the 142 piRNA clusters identified by Brennecke et al., 2007 ? Actually, the answer can be found in the legend to Figure 2. Please move this information to the main text.

We have moved the following information to the main text (Lines 129-132): “We further examined how many piRNA clusters were down- or up-regulated in *enok* mutants. Because the levels of piRNAs uniquely mapping to cluster 70, 107, 110 and 128 were zero in WT samples, these four clusters were omitted, resulting in total 138 piRNA clusters in this analysis.”

Lines 143-144: "In contrast, piRNAs mapping to RI-SL, SO-SL or het non-SL were not down-regulated in enok mutants (Fig 2B)."

Were they up-regulated as the boxplots may suggest ?

Yes, piRNAs mapping to SO-SL were slightly up-regulated in *enok* mutants. This result is similar to that in the *rhi* mutant, which also shows slight increases in levels of piRNAs mapping to SO-SL (Fig 3F in Mohn et al., 2014, Cell). We have also retrieved the small RNA-seq data of control and the *rhi* mutant from Mohn et al., 2014, and analyzed their data the same way as for our small RNA-seq data of *enok* mutants. The chart below shows that the changes in piRNA levels mapping to each group of piRNA-SL in the *rhi* mutant are similar to those in *enok* mutants (Fig 2B). This result suggests that Enok and Rhi may function in the same pathway in piRNA production.

Lines 170-172, Figure S4A:

Piwi seems to be up-regulated in enok1 mutant clones as shown in Figure S4A. This is not discussed.

We have now analyzed the RNA-seq data using edgeR (Table S4). The results showed that the expression levels of *piwi* were not significantly changed in both the *enok^1^* and *enok^2^* mutant clones.

Lines 172-173: "Immunostaining in the enok mutant germline clone ovaries also revealed reductions in the protein levels of Rhi and Mael (Fig S4C, S4D) similar to those in their mRNA levels (Fig S4A)."

Immunostaining showing one egg chamber is not sufficient to state this. Western blots are needed as a quantitative study. There is an analysis by Western blot for GFP-Rhino presented in Figure S8 but no Western-blot for Mael, in spite of the fact that anti-Mael antibodies were available.

For *enok* mutant germline clone condition (now Fig S7): Please see our response to the comment #9 from Reviewer#1.

For *enok* knockdown condition (now Fig S12): Since the mRNA levels of *mael* were unaffected in *enok* knockdown ovaries (Fig S10A; yellow bars), it is unlikely that the Mael protein level would be affected. Indeed, immunostaining of Mael shows similar levels of Mael in the control and *enok* knockdown ovaries (Fig S12A).

While the *mael* mRNA level was unaffected in *enok* RNAi-1 ovaries (Fig S10A; yellow bars), it was significantly down-regulated ~40% in *enok* mutant germline clone ovaries (Fig S7A).

Line 210:

In the results section, the authors indicate that the MTD-Gal4 driver is used for germline knock-down. The genotypes given in the figure legends do not mention MTD-Gal4. Please define MTD-Gal4 in the text to make the link with the figure legends.

We have added the genotype of MTD-Gal4 (*P{w[+mC]=otu-GAL4::VP16.R}1; P{w[+mC]=GAL4-nos.NGT}40; P{w[+mC]=GAL4::VP16-nos.UTR}CG6325^MVD1^*) in the text (Lines 236-237).

Figure 3B:

The authors should clearly indicate the sense of transcription and the promoter regions or TSS of the different genes. Moreover, there are discrepancies between the presentation of exons, introns and CDS compared to the Release 6 annotation. The legend does not indicate anything about introns, exons and CDSs. The reader can only guess what boxes and lines correspond to. The authors should verify the gene representations in this figure.

We used UCSC dm3 (Release 5) in this study as indicated in the Material and Method section. We have added the following description to the legend: “The FlyBase Genes track is shown to represent the genes, showing the exon (wider bars) and intron (blue line) structure of each annotated coding transcript, with direction of transcription indicated.”

The publication "Histone Acetyltransferase Enok Regulates Oocyte Polarization by Promoting Expression of the Actin Nucleation Factor Spire." (Fu Huang et al. Genes Dev, 28 (24), 2750-63, 2014) stated that "The expression of spir and mael are down-regulated in enok mutant ovaries". There is contradiction with the actual submitted manuscript, which concludes: "Figure S8. The protein levels of Rhi and Mael were largely unaffected in the enok knockdown ovaries."

There is essentially no discussion of this contradiction.

The original Figure S8 (now Fig S12) was performed under *enok* knockdown condition, while the previous study (Huang et al., 2014) and Figures 3A/S7A were performed in *enok* mutant germline clone ovaries. Since there were still some *enok* mRNA levels remaining in the *enok* knockdown ovaries, the effect of *enok* knockdown on *mael* expression is likely to be weaker than that of *enok* mutations. Therefore, we observed down-regulation of *mael* expression in *enok* mutant ovaries (Fig 3A and Huang et al., 2014), while the Mael level was largely unaffected upon *enok* knockdown (Fig S12).

Line 591:

Reference 3 is not correctly cited. It should be Fejes Tóth K et al.

We apologize for the error. It was generated by EndNote. We have corrected the citation.

Comments on Methods:

Normalization for RNA-seq data is not specified. Normalization to million genome-mapping reads, or all reads, whether they map to the genome or not ?

The RNA-seq reads were normalized to million genome-mapping reads.

The Drosophila Genome release used here was UCSC dm3 (Line 425, 442). Why not the latest release, which is release 6 ?

Since both the piRNA clusters and the piRNA-SL were annotated using UCSC dm3, we used dm3 in this study to incorporate the annotation of piRNA clusters and piRNA-SL retrieved from previous studies (Brennecke et al., 2007, Cell; Mohn et al., 2014, Cell).

In response to the reviewer’s concern, we have re-analyzed our RNA-seq data by edgeR using UCSC dm6. The results (edgeR-dm6) are now included in Table S4, and are very similar to the results using dm3 (edgeR-dm3).

Which TE sequences were used, from which source ?

piPipes uses two different annotations. TE sequences are from flyBase and repBase sequences are from repBase. TEtranscripts uses TE annotations from the RepeatMasker tables obtained from the UCSC genome database.

Lines 423-426: "The expression levels and the TPM values of transposon families were calculated with piPipes 1.5.0 ... (0 mismatch, multiple mappers assigned using EM algorithm) (27)".

What does that mean, random assignment of multi-mappers ?

The expectation–maximization (EM) algorithm is an approach for performing maximum likelihood estimation in the presence of latent variables. Both Cufflinks and eXpress (used in piPipes) use EM algorithm for multiple-mappers assignment.

Lines 412-413: "For examination of the mRNA levels of transposons or genes, qPCR was performed in triplicate for each sample, ..."

Are these biological (RNA extracts from different flies) or technical triplicates (from the same RNA preparation)? In the figure legends, the authors indicate that biological replicates, in general three, were used to determine p-values. Still, it is not clear whether there also were technical replicates for each RNA extract, as in common usage for qPCR, and how this was handled. Were the p-values calculated from means of technical triplicates, followed by T-tests on these means obtained for each biological replicate?

All qPCR were performed in technical triplicates for each biological replicates (as indicated in the figure legends). For example, an experiment containing 3 biological replicates would have 9 qPCR reactions. For each biological replicate, the mean of technical triplicates was used. The p-values were calculated using these means (3 means for an experiment containing 3 biological replicates) by T-tests.

Lines 426-427: "Transposon families activated in enok mutant ovaries were identified using the cutoff of P-value < 0.05, fold change > 2 and TPM > 1 in the enok1 data sets."

Why only enok1, not enok2?

We have changed to “using the cutoff of adjusted P-value < 0.05, fold change > 2 and TPM > 1 in the *enok^1^* and *enok^2^* data sets.”

How was determined this P-value, T-Test (Student)? Why didn't the authors use DESEQ analysis to identify up- or down-regulated transposon families ?

We have now used DESeq2 to identify up- or down-regulated transposon families (Table S1; TEtranscripts). TEtranscripts uses DESeq2 for differential analysis.

Line 431: "Total RNA was isolated from egg chambers of stages 5-14."

How did they isolate only stage 5-14 egg chambers ?

We dissected the ovaries and manually removed egg chambers of stages 1-4 according to the morphology. The remaining egg chambers of stages 5-14 were collected for RNA isolation. We used ovaries from germline clone females without heat-shock (never developing beyond stage 4) to compare the egg chamber morphology during dissection.

Lines 443-445: " The piRNA clusters with decreased or increased levels of piRNAs mapping to them in enok mutant ovaries were identified using the cutoff of P-value < 0.05."

Which test was used to determine these p-values?

We have now used DESeq2 to identify up- or down-regulated piRNA clusters (Table S2; adjusted P-value < 0.05).

Line 451: " P value of <2.2 x 10−16 is the maximum R can accurately calculate".

It is not clearly announced here that "R" was the tool used to calculate the p-values. Which "R" package and tool were used here?

We used “wilcox.test” in R to perform the Wilcoxon Rank sum test, and we have specified this function in the text.

Lines 449 and 481: "bins with zero reads were excluded from the analysis."

It is not sufficient to exclude bins with 0 reads to determine significant fold-change. A higher threshold, statistically defined, should be applied.

As detailed above, the original study that used this 1kb bin analysis indeed applied some cutoff criteria to define the piRNA-SL (1 kb bins). However, they did not apply any cutoff when using the 1 kb bins to analyze their NGS data. They actually added a pseudocount of 0.1 to the value of reads in each bin after normalization to library depth and mappability, in order to solve the problem that some bins would have zero reads. We instead excluded the bins with zero reads from the analysis. Therefore, we performed this 1 kb bin analysis with a more stringent criterion as compared with the original study (Mohn et al., 2014, Cell).

Line 485 " Correlation Analysis of the NGS Data"

Which data were used as input files for this correlation analysis: multiBigwigSummary or multiBamSummary, 10-kb bins genome-wide ? Please specify.

We have now specified that we used multiBamSummary, 10 kilobases by default.

Lines 501-502, Lines 511-513:

Antibodies should be denoted "anti-Piwi", "anti-Rhi" etc. for all of them, not only for "anti-H3K23ac".

We have corrected the text.

**Reviewer #3**: piRNAs have a conserved role in transposon silencing, and germline piRNAs in Drosophila are derived from heterochromatic clusters marked by the HP1 homolog Rhino. The authors present evidence that the acetyl-transferase gene enok promotes rhino gene expression and independently enhances Rhino protein binding to a subset of piRNA clusters, increasing non-canonical transcription. The findings are potentially significant, but the phenotype is complicated and the data presentation and experimental strategy are somewhat convoluted, making interpretation difficult. As a result, it is unclear if enok or H3K23 have direct or indirect roles on rhi or piRNA cluster expression. Some straightforward experiments, using tools that appear to be in hand, could directly address these points and significantly improve the manuscript.

Specific recommendations:

1. Small RNAs.

The data for transposon over-expressed in germline clones of two alleles of enok is straightforward (Figure 1), but the small RNAseq data presentation in a series of Venn diagrams obscures key details. The cutoff points (fold change and statistical significance) for "up" and "down" regulated clusters are not clearly defined in the figure legend or text. Clusters are also very complex chromosomal domains, and both transcription and piRNA production varies widely with position. A single number does not capture this complexity, or changes in the pattern of long or short RNA expression. The authors should show genome browser screen shots of piRNAs uniquely mapping to both strands of representative clusters, and transposons. Scatter plots (best) or heat maps should then be used to present the data for all clusters and all transposon families. Binned data are a good option for clusters, and bins mapping to 42AB and cluster 2 could be highlighted in a different colors. A length histogram of total piRNAs, with ping-pong z-scores for clusters and transposons would also be useful. This would allow rapid assessment of global piRNA production, visual identification of changers in expression pattern across complex clusters, and would differentiate between clusters or transposon showing modest (2 fold, for example) or profound (100 fold) changes in expression.

We thank the reviewer for these suggestions. Genome browser screen shots of piRNAs uniquely mapping to representative down-regulated (42AB and cluster 29) or unaffected (clusters 2 and 10) clusters are now shown in Figure S4B. Also, the genome browser view of piRNAs mapping to the *Burdock* transposon is included in Figure S5C, showing that the antisense piRNAs mapping to it were reduced in the two *enok* mutants.

In addition, scatter plots showing the levels of piRNAs that uniquely mapped to genic regions (left panel) and piRNA clusters (middle and right panels) in *enok* mutant germline clone ovaries versus the WT control are now added as Figure 2A. Selected piRNA clusters that were severely down-regulated in *enok* mutants (42AB, clusters 5, 29, and 78) are indicated by red dots. We have also analyzed the fold change in transposon family expression in *enok* mutants (Fig S8; y axis, enok1/WT or enok2/WT) and the fold change in antisense piRNAs encoded by the same transposon family (Fig S8; x axis, enok1/WT or enok2/WT) as performed in the previous study for the *rhi* mutant (Klattenhoff et al., 2009, Cell). The results for *enok* mutants (Fig S8) are similar to those for the *rhi* mutant (Fig S10 in Klattenhoff et al., 2009): some of the highly over-expressed transposons (*Burdock* and *HeT-A*) also show large reductions in antisense piRNAs, while expression of *blood* increases 10-12 fold but the total antisense piRNA pool is reduced by only 12%-25%. This similarity between *enok* and *rhi* mutants further supports our hypothesis that Enok may promote piRNA cluster transcription by regulating *rhi* expression and recruitment of Rhi to those clusters.

For the 1kb bin analysis, we have highlighted bins mapping to 42AB and cluster 5 (mostly down-regulated in *enok* mutants) and cluster38C1 (unaffected) in Figure S6A. Bins mapping to cluster 2 are not shown, because cluster 2 belongs to Rhi-independent source loci (RI-SL) (Mohn et al., 2014, Cell) but Figure S6A only contains Rhi-dependent source loci (RD-SL), which are further separated into Enok-dependent (ED-SL) and -independent loci (EI-SL) in this study.

The length histograms of piRNAs and pi-pong z-scores for 42AB are now shown in Figure S5A. Levels of sense and antisense piRNAs mapping to 42AB were both reduced in *enok* mutant ovaries, and the sense piRNAs were affected to a larger extent than the antisense one. The ping-pong amplification was also compromised in *enok* mutant germline clone ovaries as compared with the WT control. The newly added Figure S5B shows the length histograms of piRNAs mapping to transposons. While *enok* mutants only showed mild reductions in antisense piRNAs mapping to all transposons, antisense piRNAs mapping to some highly over-expressed transposons (*Burdock* and *HeT-A*) were more severely reduced (Fig S5C and S8).

The authors also present cluster-mapping piRNAs separated by strand, but how this was done is not defined. Clusters contain randomly oriented transposon fragments and are transcribed on both strands (42AB is typical), and genomic strand is not very meaningful for long or small RNAseq. Were cluster piRNAs broken down by strand relative to the inserted elements? If this was what was done, it needs to be stated. If not, strand separation is not very informative and piRNAs from both strands should be pooled.

The mapping of small RNA-seq reads to piRNA clusters was performed using piPipes. Also, only the unique mappers were used for piRNA clusters. Every single unique mapper only mapped to one site on the genome. Therefore, those unique piRNAs were separated by strand according to the orientation of each piRNA cluster on the genome (Watson and Crick strands). As shown in the bottom panel of Fig S5A, the sense piRNAs mapping to 42AB were affected to a larger extent than the antisense piRNAs in the two *enok* mutant germline clone ovaries. This result suggests that transcription of the sense and antisense strands in Enok-dependent clusters may show some different dependencies on Enok.

2. rhino expression/H3K27ac

RNAseq data indicates the enok mutants reduce expression of rhino, and to a lesser extent mael and BoYb. ChIPseq shows that Enok associates with the promoters for these genes, suggesting that regulation may be direct. However, the Enok ChIPseq profiles in Figure S5 suggest that this protein binds to a very large number of genes. Does binding generally correlate with changes in expression on knock down? The direct output for Enok activity is presumably H3K27 acetylation, and assaying this modification is critical to the manuscript. The authors really need to perform H3K27ac ChIPseq, and show profiles across clusters, consensus transposons, and key genes. qPCR can be used to confirm the ChIPseq data, but these assays almost certainly miss key feature. These studies should also be done in the germline clones of chromosomal mutations, not with RNAi (see below).

Indeed, Enok binds to a very large number of genes (mainly active genes). We have previously shown that Enok binds to and acetylates H3K23 at the 5’ end of *spir*, and Enok is also important for the Pol II level at *spir* 5’ in S2 cells (Huang et al., 2014, G&D). Based on these results, Enok may function as a co-activator for Pol II-dependent transcription at a subset of genes by acetylating H3K23.

Previous studies have also observed that the SAGA complex binds to a very large number of active genes (Weake et al., 2011, G&D; Li et al., 2017, G&D). The SAGA complex also functions as a co-activator for Pol II-dependent transcription. This complex contains two enzymatic modules: an acetyltransferase module that acetylates H3K9 and H3K14, and a deubiquitinase module that deubiquitinates H2B-ub. Both enzymatic activities play positive roles in gene transcription (Li et al., 2007, Cell; Wyce et al., 2007, Mol Cell). Similar to Enok, while the SAGA complex binds to a very large number of active genes, only a small fraction of genes are downregulated in SAGA mutants. Mutations in *Ada2b* disrupt the SAGA acetyltransferase activity, and global H3K9ac and H3K14ac levels are severely reduced in the *Ada2b* mutant (Pankotai et al., 2013, BMC Genomics). However, only 239 genes are downregulated in *Ada2b* mutant late L3 larvae (Pankotai et al., 2013, BMC Genomics). Based on the RNA-seq data published in Graveley et al., 2010 (available in FlyBase), ~5400 genes are expressed in late L3 larvae (cutoff: RPKM>10). Therefore, only 4.4% of the expressed genes are downregulated in the *Ada2b* mutant. In our previous study, RNA-seq analysis of *enok* mutant germline clone ovaries showed that 183 genes are downregulated in *enok* mutants, and 6732 genes are expressed in the control ovaries (cutoff: RPKM>5) (Fig S8A in Huang et al., 2014, G&D). Therefore, 2.7% of the expressed genes are downregulated in *enok* mutants. This percentage of downregulated genes in *enok* mutants is comparable to that (4.4%) in the *Ada2b* mutant.

Similarly, the *sgf11* mutant shows reduced SAGA deubiquitinase activity (Weake et al., 2008, EMBO J). In the *sgf11* mutant, only 9.5% of genes bound by SAGA are downregulated (Weake et al., 2011, G&D). This percentage of downregulated genes in the *sgf11* mutant is also comparable to those in *Ada2b* (4.4%) and *enok* (2.7%) mutants. These results suggest that many highly expressed genes may be bound by multiple co-activators, and these co-activators can compensate for each other in transcription activation to ensure robust expression. Therefore, loss of one co-activator may only affect the expression of a small subset of genes that are more dependent on that specific co-activator.

For H3K23ac ChIP-seq in the germline clones of chromosomal mutations, as the H3K23ac levels in somatic follicle cells are much higher than those in germline cells (Fig S12D), changes in H3K23ac levels in the *enok* mutant germline cells are likely to be overwhelmed by the high levels of H3K23ac in the heterozygous somatic cells in ChIP-seq. Also, *enok* mutants do not survive to adulthood, so we cannot obtain *enok* mutant ovaries in which both somatic and germline cells are mutant cells. We have successfully detected reductions in H3K23ac levels in the 5’ region of *rhi* by ChIP-qPCR using *enok* RNAi driven by both nos-Gal4-VP16 (germline driver) and tj-Gal4 (follicle cell driver) (Fig 3E). Therefore, we have subjected two independent replicates of the H3 and H3K23ac ChIP samples shown in Fig 3E to ChIP-seq analysis. Figure S13B shows the genome browser view of the H3 (control) and H3K23ac ChIP-seq results across 42AB. Consistent with the result that no Enok peaks were detected within 42AB (Fig 5A), H3K23ac levels across 42AB were largely unaffected upon *enok* knockdown (Fig S13B). In addition, the results of differential analysis on H3K23ac ChIP-seq by DESeq2 are shown in Table S6 (using H3 ChIP-seq as the normalization control). Using the cutoff of log2FoldChange< -1 and adjusted p-value<0.05, the H3K23ac levels were not significantly reduced in any piRNA clusters or transposons upon *enok* knockdown. Therefore, Enok may regulate the recruitment of Rhi to a subset of piRNA clusters including 42AB (Fig 6B) in an H3K23ac-independent way.

3. Genetics.

The reliance on RNAi knock down for many of the critical studies is puzzling, and not explained in the text. The germline clones of characterized chromosomal mutations appear to produce robust phenotypes, and are not as inherently variable as knock down. These should be used for all sequencing, with RNAi as independent verification of the data.

Indeed, the mutant germline clone ovaries appear to produce more robust phenotypes as compared with the RNAi ovaries. However, germline clones have to be generated freshly by crosses every time, and these ovaries always develop poorly (~1/5 of the normal size). As it will require 2400 and 1800 pairs of germline clone ovaries for the Pol II and Rhi ChIP-seq experiments, respectively, it is not feasible to perform ChIP-seq experiments using germline clones. Therefore, we performed RNA-seq and small RNA-seq using the *enok* mutant germline clone ovaries, and defined the Enok-dependent (ED-SL) and -independent (EI-SL) piRNA source loci using the small RNA-seq data obtained from *enok* mutants (Fig S6A). We then applied these ED-SL and EI-SL annotations in analyses on the Pol II and Rhi ChIP-seq data obtained from *enok* RNAi ovaries. Since Pol II and Rhi ChIP-seq showed that levels of Pol II and Rhi were reduced at ED-SL but not at EI-SL upon *enok* knockdown (Fig 5B and 6C), these results indeed confirmed that the data obtained from *enok* RNAi ovaries verified those obtained from *enok* mutants. Furthermore, as stated in the text, another major reason why we performed Pol II and Rhi ChIP-seq in *enok* RNAi ovaries is: we would like to show that Enok may have a role, which is independent of its role in promoting *rhi* expression, in enhancing transcription of ED-SL by facilitating Rhi recruitment. Since we showed that knocking down *enok* in the germline only mildly reduced *rhi* mRNA levels (Fig S10A; yellow bars) without affecting the protein levels of Rhi (Fig S12C-E), ChIP-seq analyses in *enok* RNAi ovaries can assess effects caused by reduced *enok* levels but with normal Rhi protein levels.

As for the ChIP-seq experiments in *enok* mutants with over-expressed *rhi*, please see our detailed response to the next comment.

4. Cluster transcription.

In Figure 4, the authors assay cluster transcription by qPCR, on RNAi knock down. However, Figure 1 presents RNAseq on germline clones of mutants. This is a much better way to define the impact of Enok on steady state cluster RNA expression, so date from the sequencing should be shown (browser shots, and scatter plots summarizing data for all clusters). Knock down and qPCR can be used to confirm the sequencing data. Similarly, Pol2 ChIPseq should be done on the germline clones. To confirm that changes in RNA and Pol2 are not due to reduced Rhino expression, the RNAseq and ChIPseq should be done in mutants over-expressing Rhino, as reported in the very nice rescue experiment in Figure S9E and F.

We thank the reviewer for the suggestion. We have analyzed the RNA-seq reads uniquely mapping to each piRNA cluster. The differential analysis by DESeq2 is now shown in Table S5, and scatter plots summarizing the RNA levels mapping to each cluster are shown in Figure S11. Six piRNA clusters (42AB, clusters 5, 29, 78, 131 and 137) are significantly down-regulated (adjusted p-value<0.05) in the two *enok* mutant germline clone ovaries (Table S5). Also, as shown in Figure S11, the four severely down-regulated clusters (42AB, clusters 5, 29 and 78; red dots) match the severely down-regulated clusters identified by small RNA-seq (Fig 2A; red dots). This result suggests that the defective transcription of a subset of piRNA clusters in *enok* mutants indeed results in reduced piRNA production from those clusters. We have also analyzed changes in RNA-seq reads uniquely mapping to ED-SL and EI-SL. As shown in the newly added Figure 5C, RNA levels at ED-SL were significantly decreased in the two *enok* mutants, while those at EI-SL were unaffected. Taken together with Figure 5B showing that Pol II levels at ED-SL were reduced upon *enok* knockdown, this result further confirms that Enok may promote transcription at these Enok-dependent piRNA source loci.

For the RNA-seq and ChIP-seq experiments in wild-type versus mutants with overexpressed *rhi* suggested by the reviewer, because *enok* mutants do not survive to adulthood, only the *enok* mutant germline clone ovaries can be used. However, germline clone ovaries always develop poorly (~1/5 of the normal size) and only 1/16 of the flies from the final cross would be the right genotype for this experiment. As a result, RNA-seq and ChIP-seq using germline clone ovaries with overexpressed *rhi* is not feasible due to difficulties to obtain sufficient materials, and that was why we performed RT-qPCR analysis shown in Figures S15E and F (originally Fig S9E-F).

5. Interpretation.

The data presented in the paper indicate that enok mutants or RNAi reduce Rhino binding and expression of a subset of piRNA clusters. Transgenic over-expression nicely demonstrates that reduced Rhino protein is not responsible for reduced binding, suggesting that Enok directly promotes Rhino binding (Figure 7D). However, the authors show that Enok does not bind to piRNA clusters and may not change H3K27ac at these domains. Rhino binding is also reduced or eliminated by mutations in del, cuff, uap56, piwi, and THO complex components, other genes are also likely to impact this process, and enok mutations appear to disrupt expression of many genes. Together, these findings suggest that lack that Enok has an indirect role in promoting Rhino binding, likely through changes in expression of other genes.

We thank the reviewer for pointing this possibility out. The expression levels of *del*, *cuff*, *uap56*, *piwi*, and the THO complex subunits (*Hpr1*, *tho2*, *thoc5*, *thoc6* and *thoc7*) were not affected in the two *enok* mutant as examined by RNA-seq (Table S4; Table S1 in Huang et al., 2014, G&D). Nevertheless, it is possible that Enok actually has an indirect role in regulating Rhi recruitment by promoting expression of other genes. We have added this possibility in the last paragraph of the Discussion section (Lines 431-433): “Therefore, it is possible that Enok may play a role in acetylating some factors that are required for Rhi recruitment, or it may have an indirect role in Rhi recruitment by promoting expression of other genes with yet unidentified functions in the piRNA pathway.”

Summary. The enok gene clearly has important functions in the germline, and histone acetylation has been largely ignored in studies on piRNA biogenesis and transposon silencing. The data presented here suggest that Enok and histone acetylation may be important for transcription of a subset of piRNA clusters. To convincingly address this possibility, and to rigorously characterize enok gene function, the authors should complete missing small RNA, long RNA, and ChIPseq experiments, using control and chromosomal mutant strains, with and without Rhino over-expression. The data should be presented and analyzed in a way that clearly summarizes the findings across the genome and transcriptome.

----------------------------------------------------------------------------------------------

We again thank the reviewers for their suggestions, which have improved the manuscript.

Sincerely,

Fu Huang, Ph.D.

Assistant Research Fellow/Professor

Institute of Biological Chemistry, Academia Sinica
