# Supplementary material for: Acetyltransferase Enok regulates transposon silencing and piRNA cluster transcription
Source: PLoS Genet. 2021 Feb 1;17(2):e1009349. doi: 10.1371/journal.pgen.1009349 (PMC7877743; doi:10.1371/journal.pgen.1009349)
Supplement: Attachment — Submitted filename: re-review_PLoS-Genetics_3aug2020.docx [file pgen.1009349.s025.docx]

PLOS Genetics

Manuscript:

"Acetyltransferase Enok regulates transposon silencing by promoting piRNA cluster

transcription"

The research topics of this manuscript are very interesting and there is a lot of data presented. After review and re-submission, the study presents much more convincing data, especially improved by new results from statistical analyses. The authors have responded to all reviewer comments and have edited the manuscript accordingly. They added new statistical results that strengthen their conclusions. Meanwhile, the study still has some methodological flaws or overstated conclusions that need to be addressed (see my comments below).

In the following comments the numbers of the lines correspond to the ones in pages 87-119 (with monitored corrections).

Results

Line 97:

The title "Transposons are activated upon loss of functional Enok in the germline" is overstated since, finally, only 7 transposons were found activated for their transcription, and in addition, only for transcription, not for any transposition activity, this was not tested here.

Lines 113-115:

Reviewer 2 comments of the 1st review process:

"Given the high degree of correlation between each independent replicate in our next generation sequencing (NGS) data sets (Fig S1 and S2), ..." "High degree of correlation" may be true for RNA-seq data (Fig. S2A), but not for the other data, where, the correlation coefficients between many replicates are quite low, 2 out of 3 coefficients being <0.90 for all small-RNA-seq replicates (Figure S2B), 1 or 2 of 3 <0.90 for Enok ChIP-seq replicates (Figure S2C), and 1 out of 4 coefficients being <0.80 for replicates from RNA Pol II ChIP-seq (Figure S2D).

answer:

Indeed, the correlation coefficients between some of our small RNA-seq or ChIP-seq (IP) replicates are between 0.85 and 0.90. Also, two coefficients between the ChIP Input replicates are between 0.75 and 0.80. However, Chan YH, 2003, Singapore Med J (44(12):614-619) defined the strength of linear relationship with a coefficient value more than 0.80 as very strong, and that with a coefficient value between 0.60 and 0.80 as moderately strong. In addition, Mukaka MM, 2012, Malawi Med J (24(3):69-71) defined the correlation with a coefficient value between 0.70 and 0.90 as high correlation (0.90-1.00 as very high). Therefore, the correlation coefficients between our NGS replicates should be able to support that the degree of correlation between them is high.

Reply reviewer 2, 2nd review:

The publication by Chan YH, 2003, Singapore Med J (44(12):614-619) does not concern NGS data but Height-Weight by Gender. This is not comparable to NGS data. Concerning the publication by Mukaka MM, 2012, Malawi Med J (24(3):69-71), entitled "Statistics corner: A guide to appropriate use of correlation coefficient in medical research", it concludes: "In summary, correlation coefficients are used to assess the strength and direction of the linear relationships between pairs of variables. When both variables are normally distributed use Pearson's correlation coefficient, otherwise use Spearman's correlation coefficient. Spearman's correlation coefficient is more robust to outliers than is Pearson's correlation coefficient." Here the authors didn't even test normality. Thus, it is not even sure that the Pearson's correlation coefficient may be used legitimately. In the publication by Mohn et al., 2014, Cell, cited by the authors as an example for their choice of statistical procedures, a publication in which NGS data are analysed, the authors observe Pearson's correlation coefficients >0.95 for NGS samples showing similarity and Pearson's correlation coefficients between 0.7 and 0.8 for non-similar NGS samples (control vs. mutant for example, Figure 4D-F). The interpretation of Pearson's correlation coefficients depends on the type of data. Other important results concerning this issue are now shown in a PCA in figure S3: As could already be suspected from the correlation coefficients in Figure S2, figure S3 clearly shows that in each case, wt, enok1 or enok2, one replicate is always apart from the other 2 replicates. The authors should have drawn consequences from this observation or they should have discussed this point in their manuscript.

Lines 131-184:

DESEQ2 analyses are very important in this section of results. They are now present in Table S2 sheet 2, but this important information is not mentioned in this paragraph. They should be indicated here since DESEQ2 analyses clearly support the other results and their conclusions in this section.

Lines 152-154:

"The ping-pong amplification of piRNAs mapping to 42AB was also compromised in enok mutant germline clone ovaries as compared with the WT control."

This is overstated because there are much less genome-unique piRNAs produced from the 42AB in the enok mutants than in wt (Table S2). Consequently, there should also be less 10-nt 5'-overlaps in the enok mutants. The z-score, if correctly calculated, is considered as significant (for a p-value <0.05), if it is >1.96. This is still the case for both enok mutants. Consequently it can not be concluded that ping-pong is compromised in the mutants. Still, I couldn't find any indication in the manuscript as to how the ping-pong z-score was determined.

Lines 167-171, Figure 2B, Table S3:

Again as already mentioned in my first review, why didn't the authors did DESEQ2 or edgeR analysis to evidence differential piRNA expression from RD-SL, as they did for piRNA clusters now ?

Lines 208-217:

reviewer 2 comments and the authors' answer of the 1st review process:

There is no link evidenced between piRNA cluster down regulation and the suggested up-regulation of 12 TE families. Actually, the authors do not show whether small RNAs complementary to these 12 TE families are down-regulated.

As the Reviewer suggested, we have used TEtranscripts (differential analysis using DESeq2) to analyze our RNA-seq data. As a result, we now only included the 7 TEs that show significant activation (adjusted p_value <0.05; Table S1) in both enok mutants as compared with the WT control in Fig 1C. We have also analyzed the fold change in transposon family expression in enok mutants (Fig S8; y axis, enok1/WT or enok2/WT) and the fold change in antisense piRNAs complementary to the same transposon family (Fig S8; x axis, enok1/WT or enok2/WT) as performed in the previous study for the rhi mutant (Klattenhoff et al., 2009, Cell). The results for enok mutants (Fig S8) are similar to those for the rhi mutant (Fig S10 in Klattenhoff et al., 2009): some of the highly over-expressed transposons (Burdock and HeT-A) also show large reductions in antisense piRNAs, while expression of blood increases 10-12 fold but the total antisense piRNA pool is reduced by only 12%-25%. This similarity between enok and rhi mutants further supports our hypothesis that Enok may promote piRNA cluster transcription by regulating rhi expression and recruitment of Rhi to those clusters.

Figure S8 doesn't show piRNA abundance but fold-change between control and enok mutant samples. Moreover, only 3 transposons, not the 7, are annotated in Figure S8. Figure S8 is not convincing to show an inverse correlation between piRNA-mapping and transcription levels. Scatterplots as in Mohn et al., 2014 Figure 4D,E would be needed also to show the level of piRNA-mapping and evidence a negative link between piRNA-mapping (small-RNA-seq) and transcription level (RNAseq) for all 7 TEs considered now.

I didn't find any similarity between the results for enok mutants in Fig. S8 presented here and those for the rhi mutant in Fig. S10 in Klattenhoff et al., 2009. A similar Figure is Fig. S9 in Klattenhoff et al., 2009. And the results in this figure are very different from those in Figure S8 presented here. Actually, In Mohn et al. Fig. S9 all transposons except 2 present fold-changes in piRNA abundance rhi/wt <1 and HeT-A piRNA abundance is reduced by 99% in rhi/wt !

Figure S9 in Klattenhoff et al., 2009:

In the present manuscript, in Figure S8, less than 40% of the transposable elements show reduced piRNA abundance (fold-change <1) and HeT-A piRNA abundance is reduced by 60-70%. Only 9 to 11 TEs show increase of expression >4.

Thus, the statement that "The enok mutants showed effects on transposon expression and piRNA abundance similar to the rhi mutants" (Figure S8), a key statement of the present manuscript, is largely overstated.

Lines 258-266:

"Six piRNA clusters (42AB, cluster 5, 29, 78, 131 and 137) were significantly down-regulated (adjusted P-value < 0.05) in the two enok mutant ovaries (Table S5)."

DESEQ2 analyses now clearly support differential transcription between enok mutants and wt for several piRNA clusters (Table S5). These are very interesting results, now supported by DESEQ2 analysis. The fact that 3 other piRNA clusters (11, 33, and 42) that show reduced piRNA levels (Fig. 2A) do not have their transcription level reduced in enok mutants is not discussed. It might be interesting for the latter piRNA clusters to examine specifically sense and antisense transcription levels since for cluster 33, only sense piRNAs levels are decreased in enok mutants and for clusters 11 and 42, only antisense piRNA levels are decreased.

Lines 416-419:

Reviewer 2 comments of the 1st review process:

Lines 358-359: "Czech et al. indeed showed that knocking down enok weakly activated the blood and Burdock transposons (less than 2-fold)." I didn't find this information about fold-change in the cited publication (ref. 23). The corresponding z-scores in this publication were -0,505 and -0,740 respectively, thus non-significant.

We apologize for this error. We have now changed the statement as follows (Lines 402-405): “Czech et al. indeed showed that knocking down enok weakly activated the blood and Burdock transposons (z-scores of -0.5 and -0.74, respectively). However, this activation effect did not reach the threshold (z-score of -1.5 or lower) applied in the screen.”

Reply reviewer 2, 2nd review:

This answer is erroneous: A z-score is considered as significant (for a p-value <0.05), if it is >1.96 ! Maybe, the authors mixed it up with the fold-change ?

The very nice Figure S4B showing the decrease of piRNAs for 42AB and piRNA cluster 29 should be shifted to a mean figure, especially as only the wt piRNA mapping for 42AB is shown in Figure 4.

Figure S4:

Fig. S4A: Y-axis: "Pais", what does that mean ? It is not specified.

Fig. S4B: The legends indicates that these are unique mappers but what is the unit, RPM, RPKM ? I suppose the read counts were normalized.

Comparison Fig 4 vs Fig S10:

The figures are very similar but the expression of the piRNA cluster transcripts are not the same, example:

cl1-A-plus/enok-RNAi-1: 0.00005 in Fig. 4B, 0.0002 in Fig. S10B

Fig. 4B:

Fig. S10B:

Why are these data presented twice, with different control samples, with "Luciferase" in Fig. 4B and with "EGFP RNAi-1" in Fig. S10B ?

Table S1 ("Table S1_TPMs of transposons.xlsx"):

Reviewer 2 comments and the authors' answer of the 1st review process:

What do values like "9,6119E-115" (FBgn0001100_G-element) and "1,4906E-188" (FBgn0020425_Helena) mean, in a table supposed to show TPM (transcripts per million) ? Moreover, it is not clear what "TPM" means. How were these values calculated?

The concept of TPM was first proposed in 2012 (Wagner et al., 2012, Theory Biosci), and it is modified from RPKM to make the average relative molar RNA concentration for each set of transcripts a constant. In this study, we used piPipes (Han et al., 2015, Bioinformatics) to calculate the TPM for each transposon family (please also see the Material and Method section).

Reply reviewer 2, 2nd review:

The problem is that such values like "9,6119E-115" (FBgn0001100_G-element) and "1,4906E-188" (FBgn0020425_Helena) are totally aberrant. Actually, if the read count is different from zero, the minimum raw read count would be 1. It cannot be less than 1. And if "1,4906E-188" corresponds to 1 read, this would mean that the library depth is 6,7086E+193 ! This is strictly impossible. If this is an error of the pipeline that the authors used here (piPipes), they should check with the developers of piPipes where the problem is and correct the values. If this is not possible, the authors should use a different pipeline and calculate the correct normalized read counts from the raw read counts if necessary. It is not possible to leave clearly aberrant results in this Table S1.

In sheet 2 of table S1, there are several satellite or other repeats (e.g. 5S_DM:RNA:RNA). The latter are not transposons (as indicated in the column title).

The transposons in sheet 1 of Table S1 are different from the transposons in sheet 2. Why is these the case, would it be possible to use the same list of transposons as in sheet 1 ?

Table S3:

1-kb bins are now shown in Table S3. To which Dm release correspond these coordinates ? Please give this information in the table.

It might be interesting to indicate the content of transposable elements for ED-SL and EI-SL.

Legend Table S4:

"The RPKM values obtained from the RNA-seq analysis of triplicate samples of the WT ovary or the enok mutants are shown (using UCSC dm3 or dm6 as the reference genome, respectively)."

If I understand well, the authors used UCSC dm3 for WT and UCSC dm6 for enok mutants. Why did they do that, especially when the aim is to compare WT and enok mutants ?

Materials and Methods:

Many data were normalized to library depth and mappability but "library depth" of the samples is not clearly defined and the method or reference of determining "mappability" could not be found in the manuscript. In Figure S1, several read counts can be found for the small-RNA-seq libraries (Total reads, aligned 0 times, aligned exactly 1 time, aligned >1 times). When analysing the "Normalized reads" in Table S3 (pooled read counts, then normalized to "library depth and mappability" as indicated by the authors), it was impossible to calculate the initial un-normalized pooled read counts using the library data given in Figure S1, neither by using "Total reads" nor the genome-unique reads ("aligned exactly 1 time"). Why is this the case ? Is there any missing information about normalization of the data ?

In their answers to reviewer comments, the authors wrote:

Table S2 shows the normalized piRNA reads assigned to each piRNA cluster as calculated by piPipes (normalized to miRNAs) (Han et al., 2015, Bioinformatics)...

This should be indicated in the Materials and Methods section and in the Table. Moreover, miRNA amounts for each library should be given in Figure S1.

Reviewer 2 comments and the authors' answer of the 1st review process:

Normalization for RNA-seq data is not specified. Normalization to million genome-mapping reads, or all reads, whether they map to the genome or not ?

The RNA-seq reads were normalized to million genome-mapping reads.

Reply reviewer 2 with respect to the revised manuscript:

It seems that this normalization to genome-mapping reads is not indicated, even in the revised manuscript.

It should be clearly indicated in the Materials and Methods sections how the data were normalized. It is not sufficient to give this information in the figure legends. Moreover the information concerning normalization should be verified throughout the manuscript to be clear and consistent with the presented data.

Lines 517-524:

Again, why did the authors pool the 3 replicates of each condition? This is not statistically sound as I already indicated in my first review. The authors answered to this major point as follows: "We have now followed the procedure described in the original study using this 1-kb bin analysis (Mohn et al., 2014, Cell) to pool the raw data from 3 replicates before normalization to library depth and mappability. The resulting processed data were then suitable for the Wilcoxon Rank sum test."

The problem is that in the cited publication by Mohn et al., 2014, only ChIP-seq replicates were pooled and this was done only because the correlation between samples was high, see the following citation from Mohn et al., 2014: "ChIP-seq libraries were mapped allowing up to three mismatches. Biological replicates were pooled after normalization to 10,000,000 mapped reads. Throughout the study, we pooled the ChIP replicates given their high correlation for all analyses." Moreover, Mohn et al. pooled the replicates after normalization, while here, the authors pooled before normalization. Thus, the procedure is not the same.

In their answers to reviewers, the authors wrote:

The expectation–maximization (EM) algorithm is an approach for performing maximum likelihood estimation in the presence of latent variables.

comment reviewer 2:

"EM" being an abbreviation, it should be defined once as "expectation–maximization (EM) algorithm" in the manuscript. This would be clear then.

In their answers to reviewers, the authors wrote:

All qPCR were performed in technical triplicates for each biological replicates (as indicated in the figure legends). For example, an experiment containing 3 biological replicates would have 9 qPCR reactions. For each biological replicate, the mean of technical triplicates was used. The p-values were calculated using these means (3 means for an experiment containing 3 biological replicates) by T-tests.

Comment reviewer 2:

It seems that the latter information for calculation of the p-values is not given in the revised manuscript.

Accessibility of the raw data:

It seems that the raw data were not accessible to reviewers, even with the password for reviewers. The message was "*External data have been provided but are not accessible for review while status is private."* The reviewers seem to have access to the description of the sequencing samples only.

Thus it was impossible to verify any of the given data of the manuscript or check reads, how many miRNAs were present in the small-RNA-seq samples for example (miRNA amount may be quite variable between small-RNA-seq samples).
