# Supplementary material for: Acetyltransferase Enok regulates transposon silencing and piRNA cluster transcription
Source: PLoS Genet. 2021 Feb 1;17(2):e1009349. doi: 10.1371/journal.pgen.1009349 (PMC7877743; doi:10.1371/journal.pgen.1009349)
Supplement: Attachment — Submitted filename: Tsai et al_Response to the reviewers comments.docx [file pgen.1009349.s026.docx]

December 28^th^, 2020

Re: PGENETICS-D-20-00022R1

Dear Drs. Chambeyron and Bickmore,

Please find below our response to the reviewers’ comments, a description of new experiments and analyses added to the manuscript, and changes to the text. We thank you for granting us the time necessary to carry out new experiments and analyses to address the reviewer’s comments. We sincerely thank the reviewers for their insightful comments. The suggested experiments and analyses have greatly helped strengthen the content and conclusions of our manuscript. Please see below a detailed discussion of these points and what we have done to address them.

---------------------------------------------------------

Reviewer #1: The manuscript was considerably improved and most of the comments were thoroughly considered. The authors present convincing evidence that Enok promotes transcription of a subset of dual-strand clusters by facilitating Rhi recruitment. My major concern is related to the interpretation of the expression and sRNAseq data for TEs. Actually, enok depletion does not robustly affect TE piRNA levels while expression of several TEs is strongly upregulated. Moreover, chromatin changes and PolII occupancy at TEs upon Enok depletion were not considered in the paper. Data related to TEs do not fit the idea that Enok is essential for the piRNA cluster transcription and primary piRNA processing and THEREFORE for TE silencing (the Title and the model in Figure 7D). This straightforward explanation should be turned down because the story of TE silencing is more complicated than direct cluster-derived piRNA-mediated cleavage of TE mRNAs. An alternative explanation should be discussed. One may suggest that Enok directly or through Rhi assistance could affect TE RNA fate despite the presence of piRNAs. Anyway, the role of Enok in TE silencing is still unclear.

Specific comments:

1. The Title should be changed to not be so causative. “Acetyltransferase Enok regulates transposon silencing AND piRNA cluster transcription” is more relevant to the reported data.

We thank the reviewer for this suggestion. We have modified the title to the suggested one.

2. The phrase (lines 148-149) “This result confirms that our small RNA-seq data are in line with the results from RNA-seq analysis (Fig 1C)” is over-statement and should be reformulate. It is the case only for Burdock and HeT-A but not for other upregulated TEs.

We have changed the phrase to “This result suggests that our small RNA-seq data are in line with the results from RNA-seq analysis (Fig 1C) for a couple of transposons including *Burdock*.”

3. In this regard, scatter plot showing the levels of all TE piRNAs in enok mutants is required. Actually, piRNA reduction could explain upregulation of a few TEs. Likely, ping-pong mechanism with participation of maternal TE-specific piRNAs masks the decreased level of primary piRNAs in enok mutants. Enhanced RNA levels of TEs in enok mutants is likely determined by another mechanism.

We have added the scatter plots showing the levels of all TE antisense piRNAs in *enok* mutants plotted against those in the WT control (Fig S8A). The seven TE families activated in *enok* mutants (Fig 1C) are marked by red dots. Indeed, the levels of antisense piRNAs mapping to *Burdock*, *HeT-A* and *Bari1* were decreased in *enok* mutants as compared with WT, while those mapping to the other 4 TE families only showed subtle reductions. We agree with the reviewer that the ping-pong mechanism involving maternal TE-specific piRNAs may mask the decreases in levels of primary piRNAs in *enok* mutants. We have added this possible explanation in the text (lines 201-204).

We thank the reviewer for this suggestion that strengthens this manuscript.

4. In the present shape, Table S1, “TEtranscripts” list, is difficult to perceive the data. It would be more clear to the readers if TEs are arranged by fold change in RNA levels. The cutoff value (e.g. 1.0-log2FC) would be suitable to mark by color the affected TEs.

We have re-arranged the “TEtranscripts” list in Table S1 as suggested by the reviewer. The significantly affected TEs are marked by purple (cutoff: adjusted P-value < 0.05, fold change > 1.5 and RPKM > 1 in mutants).

5. In Fig S4, the examples of piRNA clusters that are up-regulated in enok mutants should be present.

We have added an example of piRNA clusters that are up-regulated in *enok* mutants (cluster 73) in Figure S4B (bottom right panel).

6. Figure S7C is important to demonstrate the role of Enok in Rhi expression and would be moved to Figure 3. However, co-immunostaining of Rhi and Piwi (or some other non-affected protein) is needed to be sure that weak Rhi staining in enok mutants is not an experimental failure.

We understand the reviewer’s point. However, there is no space for Figure S7C in Figure 3. Since we emphasized more on the role of Enok in regulating the transcript levels of *rhi* in ovaries, we decided to keep Figure S7C in the original place as a piece of supportive data after careful consideration. We apologize for not able to move it to Figure 3.

Both the Rhi and Piwi antibodies are from rabbits, so Piwi is not suitable for co-immunostaining with Rhi. We have performed co-immunostaining of Rhi and Fibrillarin (Fib) in the WT control and *enok* mutant ovaries. Figure S7C is now replaced by this new data. Staining of Fib has been shown to be similar in the WT and *enok* mutant germline clone ovaries (Huang et al., 2014, G&D). Therefore, we used Fib as the staining control. We have also increased the concentration of Rhi antibody (from 1:500 to 1:200) to obtain a better staining result. As shown in the right panel in Figure S7C, Rhi signals were decreased in the *enok^1^* and *enok^2^* nurse cells, while the Fib signals remained largely unaffected. This result supports that the protein levels of Rhi are reduced upon loss of Enok in the germline.

7. Enok ChIP, Figure 3B: Negative control (no enrichment, no changes) and an example of non-piRNA pathway germline-specific gene enriched by Enok are required.

As Figure 3 has no space for more panels, we have added the two controls to the newly added Figure S9B. *CG4239* represents a negative control with no Enok enrichment and no changes in expression in *enok* mutants, and *elg1* represents a non-piRNA pathway germline-specific gene (Huang et al., 2016, G&D) enriched by Enok.

8. Figure 7D should be modified to show that Enok is essential i) for the transcription of Rhi; ii) for the Rhi-mediated transcription and primary piRNA processing of the cluster-derived piRNA precursors and iii) for TE silencing by unknown mechanism. An arrangement of arrows and brackets in this scheme, upper right, is confusing.

We thank the reviewer for this suggestion. We have modified Figure 7D to show the 3 key roles of Enok in regulating TE silencing. We have also modified the last paragraph of the result section as: “Taken together, our results suggest that Enok contributes to proper transposon silencing in the ovarian germline through three pathways summarized in Figure 7D. First, Enok acetylates H3K23 at genes involved in the piRNA production, mainly *rhi*, and promotes their expression (left). Second, Enok regulates the recruitment of Rhi to enhance transcription at piRNA clusters, facilitating the synthesis of piRNA precursors (middle). Third, as only 3 out of 7 activated transposon families (Fig 1C) showed decreased antisense piRNA levels in *enok* mutants (Fig S8A), Enok may regulate silencing of the other 4 transposons by unknown mechanisms (right).”

Reviewer #2: The research topics of this manuscript are very interesting and there is a lot of data presented. After review and re-submission, the study presents much more convincing data, especially improved by new results from statistical analyses. The authors have responded to all reviewer comments and have edited the manuscript accordingly. They added new statistical results that strengthen their conclusions. Meanwhile, the study still has some methodological flaws or overstated conclusions that need to be addressed (see my comments below).

In the following comments the numbers of the lines correspond to the ones in pages 87-119 (with monitored corrections).

Results

Line 97:

The title "Transposons are activated upon loss of functional Enok in the germline" is overstated since, finally, only 7 transposons were found activated for their transcription, and in addition, only for transcription, not for any transposition activity, this was not tested here.

We have modified the title as “Transcript levels of 7 transposons are increased upon loss of functional Enok in the germline.”

Lines 113-115:

Reviewer 2 comments of the 1st review process:

"Given the high degree of correlation between each independent replicate in our next generation sequencing (NGS) data sets (Fig S1 and S2), ..." "High degree of correlation" may be true for RNA-seq data (Fig. S2A), but not for the other data, where, the correlation coefficients between many replicates are quite low, 2 out of 3 coefficients being <0.90 for all small-RNA-seq replicates (Figure S2B), 1 or 2 of 3 <0.90 for Enok ChIP-seq replicates (Figure S2C), and 1 out of 4 coefficients being <0.80 for replicates from RNA Pol II ChIP-seq (Figure S2D).

answer:

Indeed, the correlation coefficients between some of our small RNA-seq or ChIP-seq (IP) replicates are between 0.85 and 0.90. Also, two coefficients between the ChIP Input replicates are between 0.75 and 0.80. However, Chan YH, 2003, Singapore Med J (44(12):614-619) defined the strength of linear relationship with a coefficient value more than 0.80 as very strong, and that with a coefficient value between 0.60 and 0.80 as moderately strong. In addition, Mukaka MM, 2012, Malawi Med J (24(3):69-71) defined the correlation with a coefficient value between 0.70 and 0.90 as high correlation (0.90-1.00 as very high). Therefore, the correlation coefficients between our NGS replicates should be able to support that the degree of correlation between them is high.

Reply reviewer 2, 2nd review:

The publication by Chan YH, 2003, Singapore Med J (44(12):614-619) does not concern NGS data but Height-Weight by Gender. This is not comparable to NGS data. Concerning the publication by Mukaka MM, 2012, Malawi Med J (24(3):69-71), entitled "Statistics corner: A guide to appropriate use of correlation coefficient in medical research", it concludes: "In summary, correlation coefficients are used to assess the strength and direction of the linear relationships between pairs of variables. When both variables are normally distributed use Pearson's correlation coefficient, otherwise use Spearman's correlation coefficient. Spearman's correlation coefficient is more robust to outliers than is Pearson's correlation coefficient." Here the authors didn't even test normality. Thus, it is not even sure that the Pearson's correlation coefficient may be used legitimately. In the publication by Mohn et al., 2014, Cell, cited by the authors as an example for their choice of statistical procedures, a publication in which NGS data are analysed, the authors observe Pearson's correlation coefficients >0.95 for NGS samples showing similarity and Pearson's correlation coefficients between 0.7 and 0.8 for non-similar NGS samples (control vs. mutant for example, Figure 4D-F). The interpretation of Pearson's correlation coefficients depends on the type of data. Other important results concerning this issue are now shown in a PCA in figure S3: As could already be suspected from the correlation coefficients in Figure S2, figure S3 clearly shows that in each case, wt, enok1 or enok2, one replicate is always apart from the other 2 replicates. The authors should have drawn consequences from this observation or they should have discussed this point in their manuscript.

The non-normality of NGS data has been discussed before (Mar JC, 2019, Biophys Rev, 11(1):89-94). This review also mentioned that DESeq2, CuffDiff and edgeR all use a negative binomial distribution, instead of normal distribution, for analysing RNA-seq read counts. To further confirm that our NGS data are not normally distributed, we performed normality test using the Kolmogorov–Smirnov test by R (ks.test; Mapped reads were divided into 10kb bins as we calculated the Spearman's correlation coefficients). The p-values for all our data sets are less than 0.05. Therefore, all our NGS data sets are not normally distributed, and Spearman's correlation coefficient should be suitable for the data.

Figure 4D-E in Mohn et al., 2014 only analysed piRNAs or RNAs mapping to transposons. We retrieved their small RNA-seq data and analysed the Spearman's correlation coefficients for their whole data sets:

All the correlation coefficients between *rhino*, *deadlock* and *cutoff* (showing >0.95 in their Fig 4D) are below 0.7, while the coefficients between replicates of our small RNA-seq data are above 0.86 (Fig S2).
We further calculated the Pearson's correlation coefficients for our small RNA-seq data using only the piRNA reads mapping to transposons (just like Fig 4D in Mohn et al., 2014):

We can see that data from the two *enok* mutants are more similar between each other (>=0.97) than as compared with the WT control.

Indeed, Figure S3 shows that one replicate is apart from the other 2 replicates for WT, *enok^1^* and *enok^2^*. However, all replicates from WT or *enok* mutants are still grouped together as compared with replicates from the other group (grey, pink and blue points form 3 groups without overlapping). This result suggests that, while experimental variances do exist, replicates from the same group still are more similar than between groups.

Mohn et al. (2014, Cell) mentioned in their Extended Experimental Procedures section: “Throughout the study, we pooled the ChIP replicates given their high correlation for all analyses.” Therefore, we retrieved data of their ChIP-seq replicates and calculated the Spearman's correlation coefficients:

A couple of the Spearman's correlation coefficients between their ChIP replicates are <0.84 (K9me3_ChIPseq_rhinoKD) or even <0.72 (Rhino_ChIPseq_piwiKD). Therefore, the Spearman's correlation coefficients between our NGS replicates (>0.86; Fig S2) should be able to support that the degree of correlation between them is high.

Lines 131-184:

DESEQ2 analyses are very important in this section of results. They are now present in Table S2 sheet 2, but this important information is not mentioned in this paragraph. They should be indicated here since DESEQ2 analyses clearly support the other results and their conclusions in this section.

We have now indicated that DESeq2 were used for analyses in this section: “We further examined how many piRNA clusters were down- or up-regulated in *enok* mutants using DESeq2 (Table S2).” (lines 127-128).

Lines 152-154:

"The ping-pong amplification of piRNAs mapping to 42AB was also compromised in enok mutant germline clone ovaries as compared with the WT control."

This is overstated because there are much less genome-unique piRNAs produced from the 42AB in the enok mutants than in wt (Table S2). Consequently, there should also be less 10-nt 5'-overlaps in the enok mutants. The z-score, if correctly calculated, is considered as significant (for a p-value <0.05), if it is >1.96. This is still the case for both enok mutants. Consequently it can not be concluded that ping-pong is compromised in the mutants. Still, I couldn't find any indication in the manuscript as to how the ping-pong z-score was determined.

We apologize for the confusion.
The method of calculating a z-score for the occurrence of Ping-Pong piRNA pairs were first published in Zhang et al. (2011, Mol Cell). This method, which is uninfluenced by sequencing depth, was then widely used for assessing changes in the ping-pong amplification of piRNAs. We have added the original reference in the figure legend.
The Ping-Pong z-score was calculated as follows: “For two piRNAs that were complementary to each other at a particular 5’-to-5’ distance, a score was defined as the product of their abundances. The Ping-Pong z-score was then the difference of the score at the 5’-to-5’ distance of 10 nt and the mean scores of background distances, divided by the standard deviation of the scores of background distances, defined as distances of 0–9 and 11–20 nt.” (Zhang et al., 2011, Mol Cell) Although much less genome-unique piRNAs were produced from 42AB in the *enok* mutant than in WT, both the score of 10 nt and the mean score of background distances (0-9 and 11-20 nt) were much reduced in the *enok* mutant. Therefore, the final Ping-Pong z-score would not be influenced by this defect in piRNA production from 42AB.

Also, in this original reference (Zhang et al., 2011, Mol Cell), a reduction of the Ping-Pong z-score from 20.2 to 12.5 (or 16.9 to 11.4) was interpreted as “Ping-Pong between Aub and Ago3 declined both for Aub-bound antisense piRNAs that Ping-Pong with Ago3-bound sense piRNAs and for Aub-bound sense piRNAs that Ping-Pong with Ago3-bound antisense piRNAs.” Therefore, as the z-scores for piRNAs produced from 42AB in the *enok* mutants were reduced from 12.7 (in WT) to ~3.6 (Fig S5A), we concluded that the ping-pong amplification of piRNAs mapping to 42AB was compromised in the *enok* mutants.

Lines 167-171, Figure 2B, Table S3:

Again as already mentioned in my first review, why didn't the authors did DESEQ2 or edgeR analysis to evidence differential piRNA expression from RD-SL, as they did for piRNA clusters now ?

As there are more than 6000 RD-SL (instead of only 142 piRNA clusters), DESeq2 results of each of them are difficult to perceive. In addition, the main purpose of Figure 2B is to compare the effects of losing Enok in the germline on RD-SL with the effects on other 3 groups of loci (RI-SL, SO-SL and het non-SL). Figure 2B clearly shows that loss of Enok specifically decreased the piRNA levels from RD-SL but not from other groups. DESeq2 analysis for each of these 1580-39155 loci/1kb bins would not be able to easily demonstrate this difference between RD-SL and other groups. As the result, the study that first defined these loci/1 kb bins (Mohn et al., 2014, Cell) used the method shown in our Figure 2B to analyse their NGS data and compare the results between loci groups. We followed the same method to show that loss of Enok in the germline specifically reduces piRNAs from RD-SL but not from RI-SL, SO-SL or het non-SL.

Lines 208-217:

reviewer 2 comments and the authors' answer of the 1st review process:

There is no link evidenced between piRNA cluster down regulation and the suggested up-regulation of 12 TE families. Actually, the authors do not show whether small RNAs complementary to these 12 TE families are down-regulated.

As the Reviewer suggested, we have used TEtranscripts (differential analysis using DESeq2) to analyze our RNA-seq data. As a result, we now only included the 7 TEs that show significant activation (adjusted p_value <0.05; Table S1) in both enok mutants as compared with the WT control in Fig 1C. We have also analyzed the fold change in transposon family expression in enok mutants (Fig S8; y axis, enok1/WT or enok2/WT) and the fold change in antisense piRNAs complementary to the same transposon family (Fig S8; x axis, enok1/WT or enok2/WT) as performed in the previous study for the rhi mutant (Klattenhoff et al., 2009, Cell). The results for enok mutants (Fig S8) are similar to those for the rhi mutant (Fig S10 in Klattenhoff et al., 2009): some of the highly over-expressed transposons (Burdock and HeT-A) also show large reductions in antisense piRNAs, while expression of blood increases 10-12 fold but the total antisense piRNA pool is reduced by only 12%-25%. This similarity between enok and rhi mutants further supports our hypothesis that Enok may promote piRNA cluster transcription by regulating rhi expression and recruitment of Rhi to those clusters.

Figure S8 doesn't show piRNA abundance but fold-change between control and enok mutant samples. Moreover, only 3 transposons, not the 7, are annotated in Figure S8. Figure S8 is not convincing to show an inverse correlation between piRNA-mapping and transcription levels. Scatterplots as in Mohn et al., 2014 Figure 4D,E would be needed also to show the level of piRNA-mapping and evidence a negative link between piRNA-mapping (small-RNA-seq) and transcription level (RNAseq) for all 7 TEs considered now.

We have added the scatter plots showing the levels of all transposon antisense piRNAs in *enok* mutants plotted against those in the WT control (Fig S8A), and the scatter plots of RNA-seq are in Figure 1B. Among the 7 activated transposons (Fig 1C), only 3 (*Burdock*, *HeT-A* and *Bari1*) showed reduced levels of antisense piRNAs mapping to them in *enok* mutants as compared with WT. The activation of the other 4 transposons upon loss of Enok may be mediated by another mechanism. Please also see more details in our responses to the comments #3 and #8 from Reviewer #1.

We thank the reviewer for this suggestion that strengthens this manuscript.

I didn't find any similarity between the results for enok mutants in Fig. S8 presented here and those for the rhi mutant in Fig. S10 in Klattenhoff et al., 2009. A similar Figure is Fig. S9 in Klattenhoff et al., 2009. And the results in this figure are very different from those in Figure S8 presented here. Actually, In Mohn et al. Fig. S9 all transposons except 2 present fold-changes in piRNA abundance rhi/wt <1 and HeT-A piRNA abundance is reduced by 99% in rhi/wt !

Figure S9 in Klattenhoff et al., 2009:

In the present manuscript, in Figure S8, less than 40% of the transposable elements show reduced piRNA abundance (fold-change <1) and HeT-A piRNA abundance is reduced by 60-70%. Only 9 to 11 TEs show increase of expression >4.

Thus, the statement that "The enok mutants showed effects on transposon expression and piRNA abundance similar to the rhi mutants" (Figure S8), a key statement of the present manuscript, is largely overstated.

We apologize for the confusion and for mislabelling Figure S9 as S10 for Klattenhoff et al., 2009.

Indeed, the effects of losing Enok on both piRNA levels and transposon transcript levels are weaker than those of losing Rhi, as loss of Enok only reduces (not totally eliminates) *rhi* expression by ~75% (Fig 3A). However, what we would like to show in Figure S8B (originally Fig S8) is that the different effects between *HeT-A* and *blood* is similar in both the *rhi* and *enok* mutants. As described in the above legend for Figure S9 in Klattenhoff et al., 2009, *HeT-A* showed a significant reduction in antisense piRNAs and a large increase in expression in the *rhi* mutant. On the other hand, *blood* was activated 10 fold but the antisense piRNAs were reduced by only 20%. In our Figure S8B, *HeT-A* was also significantly activated in expression and showed larger reductions (60-70%) in antisense piRNAs in the two *enok* mutants, while *blood* was activated ~10 fold but showed smaller reductions (12-25%) in antisense piRNAs.

We agree with the reviewer that the original statement is overstated. We have softened the statement: “**The results for *enok* mutants (Fig S8B) show some similarity to those for the *rhi* mutant** (15): some of the highly overexpressed transposons (*Burdock* and *HeT-A*) also showed large reductions in antisense piRNAs, while expression of *blood* increased 10-12 fold but the total antisense piRNA pool was reduced by only 12%-25%. This similarity between *enok* and *rhi* mutants further supports the hypothesis that Enok may promote piRNA biosynthesis by regulating *rhi* expression.” (lines 207-212)

Lines 258-266:

"Six piRNA clusters (42AB, cluster 5, 29, 78, 131 and 137) were significantly down-regulated (adjusted P-value < 0.05) in the two enok mutant ovaries (Table S5)."

DESEQ2 analyses now clearly support differential transcription between enok mutants and wt for several piRNA clusters (Table S5). These are very interesting results, now supported by DESEQ2 analysis. The fact that 3 other piRNA clusters (11, 33, and 42) that show reduced piRNA levels (Fig. 2A) do not have their transcription level reduced in enok mutants is not discussed. It might be interesting for the latter piRNA clusters to examine specifically sense and antisense transcription levels since for cluster 33, only sense piRNAs levels are decreased in enok mutants and for clusters 11 and 42, only antisense piRNA levels are decreased.

We apologize for the confusion. According to Brennecke et al. (2007, Cell), 91.3% of the genome unique piRNAs from cluster 33 maps to the sense strand, and 100% of the genome unique piRNAs from cluster 11 maps to the antisense strand. Since the majority of genome unique piRNAs from these two clusters maps to only one genomic strand, only sense piRNA levels or antisense piRNA levels are detected as decreased in *enok* mutants for clusters 33 and 11, respectively (Fig 2A). As for cluster 42, actually both the sense and antisense piRNA levels are decreased in *enok* mutants. We did not label cluster 42 in the middle panel (sense) in Figure 2A in the original version, and we have now added the label.

For Table S5, cluster 11 contains very low levels of unique reads from RNA-seq, and this may be why the adjusted p-value is not significant. Clusters 33 and 42 both contain an expressed gene within them, and therefore decreases in the relatively low levels of piRNA precursor transcripts may be masked by the mRNA levels from those two genes. We have added these discussions in the Result section (lines 259-264).

Lines 416-419:

Reviewer 2 comments of the 1st review process:

Lines 358-359: "Czech et al. indeed showed that knocking down enok weakly activated the blood and Burdock transposons (less than 2-fold)." I didn't find this information about fold-change in the cited publication (ref. 23). The corresponding z-scores in this publication were -0,505 and -0,740 respectively, thus non-significant.

We apologize for this error. We have now changed the statement as follows (Lines 402-405): “Czech et al. indeed showed that knocking down enok weakly activated the blood and Burdock transposons (z-scores of -0.5 and -0.74, respectively). However, this activation effect did not reach the threshold (z-score of -1.5 or lower) applied in the screen.”

Reply reviewer 2, 2nd review:

This answer is erroneous: A z-score is considered as significant (for a p-value <0.05), if it is >1.96 ! Maybe, the authors mixed it up with the fold-change ?

Czech et al. stated in their legend of Figure 2A: “Negative Z scores indicate transposon derepression.” Therefore, we interpreted z-scores of -0.5 and -0.74 as weak activation (not reaching the threshold: z-score < -1.5).

The very nice Figure S4B showing the decrease of piRNAs for 42AB and piRNA cluster 29 should be shifted to a mean figure, especially as only the wt piRNA mapping for 42AB is shown in Figure 4.

We understand the reviewer’s point. However, there is no space for Figure S4B in Figure 2. After careful consideration, we decided to keep the genome-wide results in the main figure and cluster-specific graphs in the supplementary figure. We apologize for not able to shift it to Figure 2.

Figure S4:

We believe that the reviewer means Figure S5A, so the following responses are regarding Figure S5A.

Fig. S4A: Y-axis: "Pais", what does that mean ? It is not specified.

It means the number of piRNA pairs that were complementary to each other at a particular 5’-to-5’ distance indicated by the X-axis. Reads were normalized to miRNA. We have modified the legend of Figure S5A (upper panel).

Fig. S4B: The legends indicates that these are unique mappers but what is the unit, RPM, RPKM ? I suppose the read counts were normalized.

The unit is reads normalized to miRNA. We have modified the legend of Figure S5A (lower panel).

Comparison Fig 4 vs Fig S10:

The figures are very similar but the expression of the piRNA cluster transcripts are not the same, example:

cl1-A-plus/enok-RNAi-1: 0.00005 in Fig. 4B, 0.0002 in Fig. S10B

Fig. 4B:

Fig. S10B:

Why are these data presented twice, with different control samples, with "Luciferase" in Fig. 4B and with "EGFP RNAi-1" in Fig. S10B ?

The RNA samples used in the Figure 4B were frozen and thawed once before the reverse-transcription (RT) reaction, while the RNA samples in Figure S10B were isolated and directly subjected to the RT reaction. Since the RNA levels derived from piRNA clusters are much lower than the *rp49* level (Fig 4B and S10B), these RNA levels may be affected by the freeze-thaw cycle to a more significant extent as compared with the *rp49* mRNA level. Therefore, Figure 4B shows lower RNA levels from piRNA clusters relative to *rp49*.

There are two reasons why we presented these two figures.
First, the RNAi treatment in Figure 4A-B were performed in a milder condition (at 29^o^C for 2 days) to obtain unaffected *rhi* levels in *enok* knockdown ovaries, and the RNAi treatment in Figure S10A-B were performed in a more robust condition (at 29^o^C for 3 days) to observe reduced *rhi* levels in *enok* knockdown ovaries. Figure S10A-B shows that knocking down *enok* more robustly indeed decreased *rhi* mRNA levels, further supporting that Enok promotes *rhi* expression as suggested by our RNA-seq analysis (Fig 3A). However, under milder knockdown condition, *enok* RNAi still resulted in reduced RNA levels derived from 42AB without affecting *rhi* mRNA levels (Fig 4A-B). This result suggests that Enok may have roles, other than promoting *rhi* expression, in regulating transcription at 42AB, and therefore we further discovered that Enok is also important for the recruitment of Rhi to 42AB in Figures 6-7.
Second, we also would like to show more convincingly that the effects of *enok* knockdown are reproducible using two different control strains (Luciferase and EGFP RNAi-1). Similarly, these two different control strains were used in Figures 7A-B and S15C-D.

Table S1 ("Table S1_TPMs of transposons.xlsx"):

Reviewer 2 comments and the authors' answer of the 1st review process:

What do values like "9,6119E-115" (FBgn0001100_G-element) and "1,4906E-188" (FBgn0020425_Helena) mean, in a table supposed to show TPM (transcripts per million) ? Moreover, it is not clear what "TPM" means. How were these values calculated?

The concept of TPM was first proposed in 2012 (Wagner et al., 2012, Theory Biosci), and it is modified from RPKM to make the average relative molar RNA concentration for each set of transcripts a constant. In this study, we used piPipes (Han et al., 2015, Bioinformatics) to calculate the TPM for each transposon family (please also see the Material and Method section).

Reply reviewer 2, 2nd review:

The problem is that such values like "9,6119E-115" (FBgn0001100_G-element) and "1,4906E-188" (FBgn0020425_Helena) are totally aberrant. Actually, if the read count is different from zero, the minimum raw read count would be 1. It cannot be less than 1. And if "1,4906E-188" corresponds to 1 read, this would mean that the library depth is 6,7086E+193 ! This is strictly impossible. If this is an error of the pipeline that the authors used here (piPipes), they should check with the developers of piPipes where the problem is and correct the values. If this is not possible, the authors should use a different pipeline and calculate the correct normalized read counts from the raw read counts if necessary. It is not possible to leave clearly aberrant results in this Table S1.

We agree with the reviewer. We have now used RPKM in Figure 1C and Table S1.

In sheet 2 of table S1, there are several satellite or other repeats (e.g. 5S_DM:RNA:RNA). The latter are not transposons (as indicated in the column title).

We agree with the reviewer that there are some satellites or repeats in the list. We showed results of all transposons, satellites and repeats from TEtranscripts analysis to avoid any bias or suspicion of hiding results.

The transposons in sheet 1 of Table S1 are different from the transposons in sheet 2. Why is these the case, would it be possible to use the same list of transposons as in sheet 1 ?

We are sorry that the two different analysis packages (piPipes and TEtranscripts) showed results with similar but slightly different lists. While piPipes can compare expression levels of transposons, noncoding RNAs and mRNAs using RNA-seq data (Fig 1B), TEtranscripts analyses changes in transposon expression using DESeq2 (Fig 1C and Table S1). All the transposons that are significantly activated (adjusted p_value<0.05; Fold change>1.5; RPKM>1 in mutants) in *enok* mutants as identified by TEtranscripts (sheet 2 in Table S1) are also included in sheet 1. Therefore, the results from piPipes (sheet 1) and TEtranscripts (sheet 2) should be compatible.

Table S3:

1-kb bins are now shown in Table S3. To which Dm release correspond these coordinates ? Please give this information in the table.

The 1-kb bins are annotated using Dm3. We have added this information in Table S3.

It might be interesting to indicate the content of transposable elements for ED-SL and EI-SL.

We have indicated the names of transposon sequences in each 1-kb bin in Table S3.

Legend Table S4:

"The RPKM values obtained from the RNA-seq analysis of triplicate samples of the WT ovary or the enok mutants are shown (using UCSC dm3 or dm6 as the reference genome, respectively)."

If I understand well, the authors used UCSC dm3 for WT and UCSC dm6 for enok mutants. Why did they do that, especially when the aim is to compare WT and enok mutants ?

We apologize for the error in writing. We mean that we used dm3 and dm6 for both WT and *enok* mutants. Therefore, there are two sheets in Table S4: “edgeR-dm3” and “edgeR-dm6”. Both sheets contain data from WT and *enok* mutants. We have deleted the word “respectively” from the legend.

Materials and Methods:

Many data were normalized to library depth and mappability but "library depth" of the samples is not clearly defined and the method or reference of determining "mappability" could not be found in the manuscript. In Figure S1, several read counts can be found for the small-RNA-seq libraries (Total reads, aligned 0 times, aligned exactly 1 time, aligned >1 times). When analysing the "Normalized reads" in Table S3 (pooled read counts, then normalized to "library depth and mappability" as indicated by the authors), it was impossible to calculate the initial un-normalized pooled read counts using the library data given in Figure S1, neither by using "Total reads" nor the genome-unique reads ("aligned exactly 1 time"). Why is this the case ? Is there any missing information about normalization of the data ?

The reference for the mappability of each 1-kb bin is Mohn et al. 2014, as they provided both the coordination and mappability of all 1-kb bins used in this study. We have added this reference in the Material and Methods section. We have now also indicated the definition of “library depth” for each case.

In their answers to reviewer comments, the authors wrote:

Table S2 shows the normalized piRNA reads assigned to each piRNA cluster as calculated by piPipes (normalized to miRNAs) (Han et al., 2015, Bioinformatics)...

This should be indicated in the Materials and Methods section and in the Table. Moreover, miRNA amounts for each library should be given in Figure S1.

We have modified the Materials and Methods section : “The abundance of piRNAs mapping to each piRNA cluster or genic region shown in Table S2 were calculated with piPipes 1.4.12, using the default small RNA-seq pipeline with UCSC dm3 as the reference genome (0 mismatches; only unique mappers; normalized to miRNAs).” In the Table legend, this information is also provided: “The unique reads mapping to each piRNA cluster or genic region were normalized to miRNAs and defined as the abundance of piRNAs.” We have also added the number of miRNA reads for each small RNA-seq library in Figure S1.

Reviewer 2 comments and the authors' answer of the 1st review process:

Normalization for RNA-seq data is not specified. Normalization to million genome-mapping reads, or all reads, whether they map to the genome or not ?

The RNA-seq reads were normalized to million genome-mapping reads.

Reply reviewer 2 with respect to the revised manuscript:

It seems that this normalization to genome-mapping reads is not indicated, even in the revised manuscript.

We have added this information in the Material and Methods section (line 489).

It should be clearly indicated in the Materials and Methods sections how the data were normalized. It is not sufficient to give this information in the figure legends. Moreover the information concerning normalization should be verified throughout the manuscript to be clear and consistent with the presented data.

We thank the reviewer for this advice. We have verified that we have provided clear and consistent information regarding normalization in this manuscript.

Lines 517-524:

Again, why did the authors pool the 3 replicates of each condition? This is not statistically sound as I already indicated in my first review. The authors answered to this major point as follows: "We have now followed the procedure described in the original study using this 1-kb bin analysis (Mohn et al., 2014, Cell) to pool the raw data from 3 replicates before normalization to library depth and mappability. The resulting processed data were then suitable for the Wilcoxon Rank sum test."

The problem is that in the cited publication by Mohn et al., 2014, only ChIP-seq replicates were pooled and this was done only because the correlation between samples was high, see the following citation from Mohn et al., 2014: "ChIP-seq libraries were mapped allowing up to three mismatches. Biological replicates were pooled after normalization to 10,000,000 mapped reads. Throughout the study, we pooled the ChIP replicates given their high correlation for all analyses." Moreover, Mohn et al. pooled the replicates after normalization, while here, the authors pooled before normalization. Thus, the procedure is not the same.

We apologize for the mistake. We have re-analysed our NGS data to follow the procedure described in Mohn et al., 2014 correctly, and have updated all figures showing the 1-kb bin analysis with the new results. All the new results still support our original conclusions. We have also updated the Materials and Methods section: “For the 1 kb bin analysis, only the unique mappers were used. The definition of RD-SL, RI-SL, SO-SL and het non-SL loci was obtained from the previous study (14). After normalized to library depth (uniquely mapped reads), the mapped reads from 3 independent replicates were pooled and normalized to mappability (14), and bins with zero reads were excluded from the analysis.”

As for the correlation between samples, a couple of the Spearman's correlation coefficients between the ChIP replicates in Mohn et al., 2014 are <0.84 (K9me3_ChIPseq_rhinoKD) or even <0.72 (Rhino_ChIPseq_piwiKD) (please see our above detailed responses regarding correlation). Therefore, the Spearman's correlation coefficients between our NGS replicates (>0.86; Fig S2) should be able to support that the degree of correlation between them is high.

In their answers to reviewers, the authors wrote:

The expectation–maximization (EM) algorithm is an approach for performing maximum likelihood estimation in the presence of latent variables.

comment reviewer 2:

"EM" being an abbreviation, it should be defined once as "expectation–maximization (EM) algorithm" in the manuscript. This would be clear then.

We have indicated this full name in the manuscript (line 489).

In their answers to reviewers, the authors wrote:

All qPCR were performed in technical triplicates for each biological replicates (as indicated in the figure legends). For example, an experiment containing 3 biological replicates would have 9 qPCR reactions. For each biological replicate, the mean of technical triplicates was used. The p-values were calculated using these means (3 means for an experiment containing 3 biological replicates) by T-tests.

Comment reviewer 2:

It seems that the latter information for calculation of the p-values is not given in the revised manuscript.

We have added this information in the manuscript (lines 480-481).

Accessibility of the raw data:

It seems that the raw data were not accessible to reviewers, even with the password for reviewers. The message was "*External data have been provided but are not accessible for review while status is private."* The reviewers seem to have access to the description of the sequencing samples only.

Thus it was impossible to verify any of the given data of the manuscript or check reads, how many miRNAs were present in the small-RNA-seq samples for example (miRNA amount may be quite variable between small-RNA-seq samples).

We are sorry that we need to set the data sets as private in GEO before this manuscript is published. While GEO provides reviewer token for private sessions, it seems that is all GEO shows to the reviewers. There is not much we can do to improve it. We will set the data sets as public once this manuscript is accepted. We will definitely retract this manuscript if people find that our analytic results cannot be reproduced from the raw data.

As suggested by the reviewer, we have added the number of miRNA reads for each small RNA-seq library in Figure S1. The miRNA amount [the (miRNA/total mapped reads) ratio] in each library is similar to each other (16%-21%).

----------------------------------------------------------------------------------------------

We again thank the reviewers for their suggestions, which have improved the manuscript.

Sincerely,

Fu Huang, Ph.D.

Assistant Research Fellow/Professor

Institute of Biological Chemistry, Academia Sinica
